# Ontogenetic variation in the skull of *Stenopterygius quadriscissus* with an emphasis on prenatal development

**DOI:** 10.1038/s41598-022-05540-0

**Published:** 2022-02-01

**Authors:** Feiko Miedema, Erin E. Maxwell

**Affiliations:** 1 Staatliches Museum Für Naturkunde Stuttgart, Rosenstein 1, 70191 Stuttgart, Germany; 2 Department of Paleontology, Hohenheim University, Schloss Hohenheim 1 A, 70599 Stuttgart, Germany

**Keywords:** Palaeontology, Bone development, Embryology, Herpetology

## Abstract

The availability of a large sample size from a range of ontogenetic stages makes *Stenopterygius quadriscissus* a good model to study ontogenetic variation in a fossil sauropsid. We qualitatively examined pre- and postnatal ontogenetic changes in the cranium of *S. quadriscissus*. The prenatal ossification sequence is similar to other diapsids, exhibiting delayed chondrocranial ossification compared to the dermatocranium. In the dermatocranium, the circumorbital area is more ossified earlier in development relative to other elements, especially those of the skull roof where ossification is comparatively weaker across prenatal stages. Perinatally all cranial elements are ossified, and many scarf and step joints are already closed. We propose four prenatal and three postnatal stages in *S. quadriscissus* on the basis of relative ossification, size and qualitative cranial characters pertaining to the jugal, parietal, frontal, pterygoid and surangular. These will provide a basis for determining ontogenetic stages in other ichthyosaurs. Moreover, our postnatal observations aid in refining ontogenetic characters for phylogenetic studies. Lastly, we observed that the antimeric sutures of the midline of the skull roof are open perinatally and that fusion of the midline only appears in the adult stage. We hypothesize that the loose connection of the midline functions as a fontanelle, limiting potential damage during birth.

## Introduction

Ontogeny is a major factor controlling ossification and morphology. In extinct diapsids, the small number of specimens in most taxa hinders assessment of ontogenetic variation. However, ontogenetic assessment is necessary for understanding paleobiology and refining phylogenetic characters. A few species preserve a wide range of ontogenetic stages, which makes them key in studying developmental evolution in extinct clades. One of these is the parvipelvian ichthyosaur *Stenopterygius quadriscissus*. Like all ichthyosaurs *S. quadriscissus* is viviparous and many pregnant individuals of this taxon have been found, enabling us to study its prenatal development. Studies have examined ontogenetic allometry between major body regions^1^ and the apparent reduction in the tooth crown height over ontogeny and subsequent functional edentulism^2^, which correlated with trophic differences between juveniles and adults^3^. Lastly, ontogenetic variation in this species has been qualitatively assessed in the braincase and limb, including ossification and integration^4–8^. Substantial differences in the forelimb between juveniles and adults of *S. quadriscissus* were observed in the shape of the proximal articular surface of the humerus, the surface texture of the humerus, the prevalence of notching of the elements of the leading edge of the limb and the suture closure between individual limb elements^4^. A proximal–distal ossification gradient in the fore- and hindlimb, and early ossification in the girdle elements have also been described, with the ossification of both girdles preceding the limbs and the ossification of the pectoral girdle preceding the pelvic girdle^6^. In the braincase, notable ontogenetic changes were observed in e.g. the size and shape of the basioccipital peg, the morphology and relative size of the foramen for the internal carotid artery on the parabasisphenoid and the width of the impression of the semicircular canals on the prootic^5^. In this study we add to the current knowledge of ontogenetic variation in *S. quadriscissus* by describing changes in morphology of the dermal skull, articular and hyoid apparatus. Furthermore, we propose 4 prenatal and 3 postnatal ontogenetic stages on the basis of cranial ossification. We compare the morphologies to the ontogenies of related taxa and discuss if characters based on the elements discussed currently used in phylogenetic analyses are ontogenetically variable in *S. quadriscissus*. This improves knowledge of the osteological development of *Stenopterygius* and refines our understanding of variability in osteological characters used in ichthyosauromorph cladistic analyses. We also propose terminology for ontogenetic stages in Neoichthyosauria both prenatal and postnatal based on the ontogenetic variation in the cranium of *S. quadriscissus*.

## Methods

We examined ~ 30 specimens of the genus *Stenopterygius* from all possible ontogenetic stages at the Staatliches Museum für Naturkunde Stuttgart (SMNS), as well as comparative material at the Urweltmuseum Hauff, Holzmaden (MHH). All specimens are from the Early Jurassic Posidonienschiefer Formation. Almost all morphology discussed is based on specimens assigned to *Stenopterygius quadriscissus* following the metrics used by Ref.^9^. Most of the ontogenetic variation discussed is therefore intraspecific. In the cases where observations in specimens of *S. triscissus* or indeterminate material of *Stenopterygius* are noted, this is stated clearly in the text and figures. As *Stenopterygius* does not display lines of arrested growth (LAGs), histology was not an option to subdivide specimens into ontogenetic stages based on absolute specimen ages^10^. We subdivided postnatal juvenile and adult stages on the basis of the length of the lower jaw in the smallest gravid female^11^ and qualitatively assessed differences between specimens in ossification based on bone surface texture (e.g., rugose/smooth), the level of fusion of sutures and morphological differences. Embryonic stages are likewise based on degree of relative cranial ossification (discussed in detail in results and comparison), mostly changes in surface texture and element–element contact. Due to the most common mode of preservation in the Posidonienschiefer Formation (slab-mounted specimens) elements were usually only visible in one view per specimen, which sometimes hampered comparing different views of elements over ontogeny. However, for most elements the sample size was high enough to capture the most important morphological changes. We use disarticulated material whenever possible. *Stenopterygius*, like all other members of Ichthyosauria, has many scarf and step joints as cranial sutures (sensu^12^), especially in the circumorbital and cheek regions. Only observing articulated specimens would therefore hamper our assessment of the variation in most elements. Photographs were taken in standard indoor light conditions. We used a 105 mm macro-lens to photograph embryonic material.

### Terminology

Recently, there has been an attempt to standardize ontogenetic terminology and best practice regarding assessing ontogenetic effect in extinct reptiles, emphasizing the need for broadly used, well-defined terminology^13^. In this paper we propose postnatal ontogenetic stages within *S. quadriscissus* based not only on mandibular length but also on cranial ossification and morphology. Justification for these groups and discussion of morphology will be given in the results; however we propose three postnatal stages: postnatal 1, which are in general smaller juveniles (single individuals with mandible length < 300 mm); postnatal 2, which are large juveniles and those sometimes referred to as subadults (mandible length 300–399 mm); and sexually mature individuals, which include all specimens with a mandible length longer than or equal to the smallest pregnant female (400 mm). For clarity we already use these terms throughout the description and results.

#### Prenatal ossification stages

Most pre-natal ichthyosaurian material has been referred to generically as ‘embryonic’ (used to comply with sauropsid terminology) or ‘fetal’ (used to comply with viviparous terminology, which originates in mammalian biology)^14–17^. Attempts to infer the developmental stage of two embryos associated with specimens of *Ichthyosaurus somersetensis* used the ratios of orbital size and skull length as a proxy for developmental time and comparing these measures to those of embryos of *Alligator mississippiensis*^18^. Based on this ratio, the embryos were considered to be at a comparable stage to stages 16 and 19/20 respectively of *A. mississippiensis*^18,19^. This approach assumes a similar allometric trajectory in *Alligator* and *Ichthyosaurus*; however, ichthyosaurs retain proportionately larger orbits in adults^20^, making this approach inappropriate and underestimating relative ontogenetic stage. We use ossification as a more reliable comparative metric.

**Figure 1:**
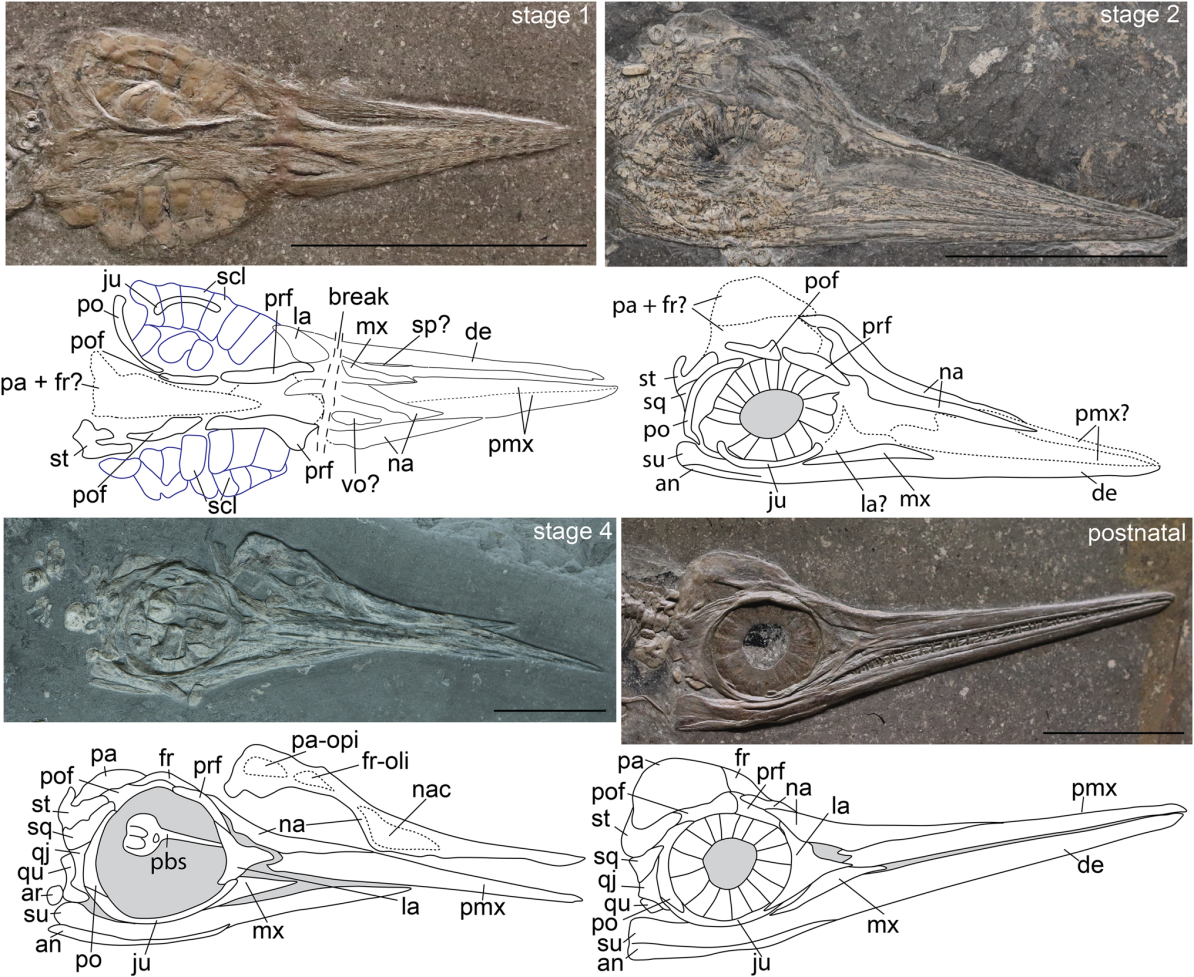
Proposed prenatal stages and earliest postnatal developmental stage of *Stenopterygius quadriscissus* on the basis of cranial ossification of single elements and suture closure. Stage 3 is subdivided into 3a and 3b on the basis of ossification of isolated elements and only represented by disarticulated material. Stage 1, SMNS 10460; Stage 2, SMNS 50963; Stage 4, SMNS 54064; Postnatal 1, SMNS 12821. *an* angular, *ar* articular, *de* dentary, *fr* frontal, *fr-oli* frontal olfactory lobe indentation, *ju* jugal, *la* lacrimal, *mx* maxilla, *na* nasal, *nac* nasal capsule, *pa* parietal, *pa-opi* parietal optic lobe indentation, *pmx* premaxilla, *po* postorbital, *pof* postfrontal, *prf* prefrontal, *qj* quadratojugal, *qu* quadrate, *scl* sclerotic plate, *sp* splenial, *sq* squamosal, *st* supratemporal, *su* surangular, *vo* vomer. Scale bar (all parts) = 50 mm.

In *Alligator*, cranial ossification begins around stage 19^19,21^. Albeit still weakly ossified, the *Ichthyosaurus* embryos^18^ clearly have a higher degree of ossification in the circumorbital region than stage 19 *Alligator*, suggesting a later stage. All studied embryos of *S. quadriscissus* have either the same or a higher level of ossification than the studied *Ichthyosaurus somersetensis* specimen. Differences in ossification between the studied embryonic specimens of *S. quadriscissus* allow us to recognize four potential stages of embryonic development, defined on the basis of the degree of cranial ossification (Fig. 1). The earliest stage is represented by three embryos associated with SMNS 10460 (two of which are preserved well-enough to assess ossification). The second stage is represented by two (or possibly just one) embryos associated with SMNS 50963. These early developmental “stages” were previously hypothesized based on girdle elements^6^ and size^22^. The third stage is represented by isolated embryonic elements associated with SMNS 80234. In this specimen, there are ossification differences between embryonic elements associated with the same female, as noted earlier in the braincase^5^. We therefore split stage 3 into “a” and “b”. The last stage recognized is the perinatal stage (stage 4), represented by various embryos associated with different specimens (most notably: SMNS 54064, SMNS 54062, SMNS 16811, SMNS 81961, SMNS 52036 and SMNS 6293). The developmental stages we recognize in this paper are based only on cranial ossification. The stages recognized are defined only relative to each other, and therefore do not attest to precise timing of development and cannot be directly correlated with other sauropsid ossification-based staging systems at present. Ossification centres were identified based on an outwardly radiating pattern of bone fibres from a single point in prenatal stages.

## Results

The results are subdivided into two parts. In the first section, we describe the different prenatal stages and their relative ontogenetic differences. In the second section we describe the relevant ontogenetic changes throughout pre- and postnatal ontogeny of each element separately. The results are organized in this way to show the different prenatal ontogenetic stages specifically and the phylogenetically relevant ontogenetic changes per element. Lastly, we summarize the major and phylogenetically relevant ontogenetic variation.

### Description of prenatal stages

#### Stage 1

Overall ossification in this stage is very poor (Fig. 1). The rostral elements are visible but weakly ossified; most strongly in the posterior area of the nasal. The frontal and parietal are present, but largely unossified. The best ossified elements are the circumorbital elements: the jugal, postorbital, postfrontal and prefrontal which, although relatively well ossified, maintain broadly open scarf and step sutures. The posterior part of the nasal is likewise well-ossified. The postorbital is relatively large. The postfrontal is well ossified but lacks its distinct anterior expansion containing the frontal facet. The circumorbital portion of the prefrontal is likewise better ossified than the remainder of the element. The lacrimal appears less well ossified than the other circumorbital elements. The supratemporal is relatively well ossified, displaying clear medial and lateral rami. The squamosal and quadratojugal are present but do not share the same degree of ossification as the supratemporal. It is likely some palatal elements (possibly the vomer) have started ossification. The mandibular elements that are visible in lateral view are present but not well ossified. There is no indication of an articular (although this element is easily hidden by others). Likewise, there is no indication of ossified braincase elements except for the parabasisphenoid (Supplementary Fig. 1 and accompanying discussion S1). The sclerotic plates are as large as or larger than most ossified cranial elements and are very well ossified. The orbital diameter is equal to the length of the rostrum.

#### Stage 2

Overall ossification state in stage 2 is slightly higher than in stage 1. The circumorbital elements remain the most prominently ossified elements. The postorbital remains relatively large, but is very curved and has no apparent facets. The anterior expansion of the postfrontal is present, although it remains relatively small. The nasal is better ossified than in stage 1, especially the rostral and internarial areas. The lacrimal remains poorly ossified. The jugal is hidden by the scleral plates in the specimen examined, but is clearly ossified, causing deformation of the ventral edges of the sclerotic plates. The squamosal and likely quadratojugal are in articulation ventral to the supratemporal. The parietal and frontal remain relatively weakly ossified. However, the posterior portions of the parietals have seemingly started ossification. Palatal elements are visible in the orbit in the examined specimen, likely the palatal rami of the pterygoids and/or the cultriform process of the parabasisphenoid. The rostral elements are better ossified than in stage 1 and the rostrum has lengthened relative to the diameter of the orbit. Teeth have started mineralization. As with the upper jaw elements, the mandibular elements are better ossified than in stage 1. There is a clear suture visible between the surangular and angular posteriorly. The articular is not observed, but is possibly hidden in the examined specimen. The sclerotic plates remain the most prominent ossified elements, and are very large compared to other cranial elements.

#### Stage 3

All cranial elements are well ossified. Due to the disarticulated nature of the specimen representing this stage, it is difficult to compare directly with articulated material. However, this stage is intermediate between stages 2 and 4 (close to birth), based on the degree of ossification in certain elements (e.g., parietal, supratemporal and basisphenoid). It has previously been noted that this specimen likely consists of two different ossification stages based on braincase elements^5^; the same phenomenon affects the parietals (Fig. 5A,B). However, most other cranial elements do not show variation in ossification. The rostral, mandibular and circumorbital elements have the highest degree of ossification, whereas the palate, skull roof and braincase elements are least ossified. Ossification of all braincase elements to the point where individual elements can be recognized in disarticulated material has begun by this stage^5^. An element tentatively identified as the articular (Supplemental Fig. 10A) is still in early stages of ossification. The morphology of the elements of the skull roof, circumorbital area, mandible and rostrum resemble postnatal stages.

#### Stage 4 (perinatal)

The degree of ossification is higher than in stage 3 and all elements display their postnatal articulation (Fig. 1) and most their postnatal morphology. The orbit remains relatively large compared to the sexually mature stage, but the rostrum has lengthened relative to the orbit compared to earlier stages. The numerous scarf and step joints in the cheek and circumorbital areas of the cranium have closed, but with clearly visible suture lines. The butt joints along the midline of the cranium remain open.

### Note on ossification sequence

The timing and sequence of onset of ossification cannot be assessed in our sample. However, general trends are clear. The bones in the circumorbital area are ahead in terms of ossification throughout stages 1 through 3, followed by the cheek area and the dermal portion of the lower jaw (i.e., every element except the articular). The rostral elements, with the exception of the maxilla are weakly ossified relative to the circumorbital region in stages 1 and 2, but have seemingly caught up by stage 3. The skull roof, especially the parietal and frontal, lag behind the circumorbital, lower jaw and rostral areas in ossification up to stage 4. The braincase, palatal elements, and articular are only observed in stages 3 and 4. This does not mean that they could not be present in stage 1 and 2, as these elements are situated internally. However, the state of ossification of these elements is fairly low compared to circumorbital or even skull roof elements in stage 3, and they do not show juvenile morphology in the perinatal stage, unlike the circumorbital, rostral and dermal lower jaw elements. This leads to the deduction that their ossification likewise lags behind that of non-palatal dermatocranial elements. Delayed chondrocranial ossification is also seen in extant reptiles^21,23–28^. The sequence of dermatocranial ossification is variable between taxa, but the skull roof often lags behind most other dermatocranial elements in ossification in sauropsids. Interestingly in at least some extant amphibians, delayed chondrocranial ossification is not recorded, nor does the frontoparietal show delayed ossification^29,30^. Cranial development of *Stenopterygius quadriscissus* therefore seems to be consistent with other diapsids.

### Per element assessment of relevant prenatal and postnatal ontogenetic changes

#### Antorbital rostrum

##### Premaxilla

The premaxilla is often preserved in lateral view. The subnarial process usually contacts the lacrimal in a butt-joint (thereby excluding the maxilla from the external naris); however, contact between the lacrimal and premaxilla is variable in *Stenopterygius*^31^. The supranarial process overlaps the nasal presumably in a step joint.

The premaxilla is ontogenetically conservative, as also noted in *Platypterygius australis*^32^. Ossification is already fairly advanced in embryonice stage 3 (Supplemental Fig. 2A). In lateral view, the premaxillary fossa is possibly present in embryonic stage 2 and apparent in embryonic stage 4, the supra-and subnarial processes likewise resemble the sexually mature morphology (Supplemental Fig. 2D–G). In ventral view, the walls of the alveolar groove are more weakly developed and the groove appears wider than in the sexually mature stage (Supplemental Fig. 2A–C). The maxillary and vomerine rami are distinct in all stages as the maxillary ramus is slightly offset, whereas the vomerine ramus is directed in line with the anterior part of the premaxilla. However, they often appear separate from the rest of the element in embryonic stage 3, likely due to the fact that the midsection is less well ossified than the margins in this view (Supplemental Fig. 2A).

##### Nasal

The nasal has two broad flanges posteriorly, visible in lateral or dorsal view and separated by a convex dorsal ridge lateral to the excavatio internasalis (Supplemental Fig. 3E).

The nasal is ontogenetically conservative. It is well ossified in embryonic stage 3, but less so than the dermatocranial lower jaw elements. There is a clear ossification centre dorsal to the lateral ramus (Supplemental Fig. 3A,F). In embryonic stage 3 the lateral ramus is more prominent, whereas the medial flange is relatively smaller (Supplemental Fig. 3D). In embryonic stage 4, both flanges are similarly developed (Supplemental Fig. 3B). In both these stages there is no thickening of the convex ridge separating the lateral and dorsal flanges. It was difficult to assess ontogenetic variation in the excavatio internasalis due to taphonomic compression. As morphology does not change very much between postnatal stages, we assume that the excavatio internasalis was at least present. The posteromedial nasal has a distinct facet on its ventral surface in embryonic stage 3 for contact with the frontal (Supplemental Fig. 3A). All other nasals are either exposed in dorsal view, or tightly sutured to the frontal hindering assessment of the development of this facet. From prenatal stage 4 onwards, the nasal displays a prominent central depression just anterior to the external naris, which we interpret as the accommodation for the nasal capsule (Supplemental Fig. 3A–C). Also visible on the ventral side of the stage 4 embryonic nasal is an anteroposteriorly elongated plate protruding ventrally from the nasal capsule area representing the developing vomerine process. There is virtually no difference other than size between the postnatal 2 and sexually mature stages in dorsal and lateral view (Supplemental Fig. 3C,E,G).

##### Maxilla

The maxilla is overlapped by the lacrimal, jugal, and premaxilla; medially it contacts the palatine. The ventral surface of the maxilla forms the alveolar groove, and is dentigerous.

In embryonic stage 3 and to a lesser degree in the perinatal and postnatal 1 stages, the posterior margin of the maxilla is split into two distinct rami, which disappear or merge into the element in the sexually mature or postnatal 2 stage (see Supplemental Fig. 4A–D). The dorsomedial and most elongated of the two is likely the ramus that bears the jugal facet. This facet is clearly visible in the sexually mature stage as a groove and is likely also present in earlier ontogenetic stages. The ventrolateral of the two rami is either the ventral edge of the jugal ramus/facet or the posterior extent of the tooth row. In embryonic stage 3, a second anteriorly directed ramus was observed. This is possibly the medial wall of the maxilla, which contacts the palatine. It is difficult to determine its true identity since maxillae are most often preserved in lateral view and in articulation.

#### Circumorbital area

##### Lacrimal

**Figure 2:**
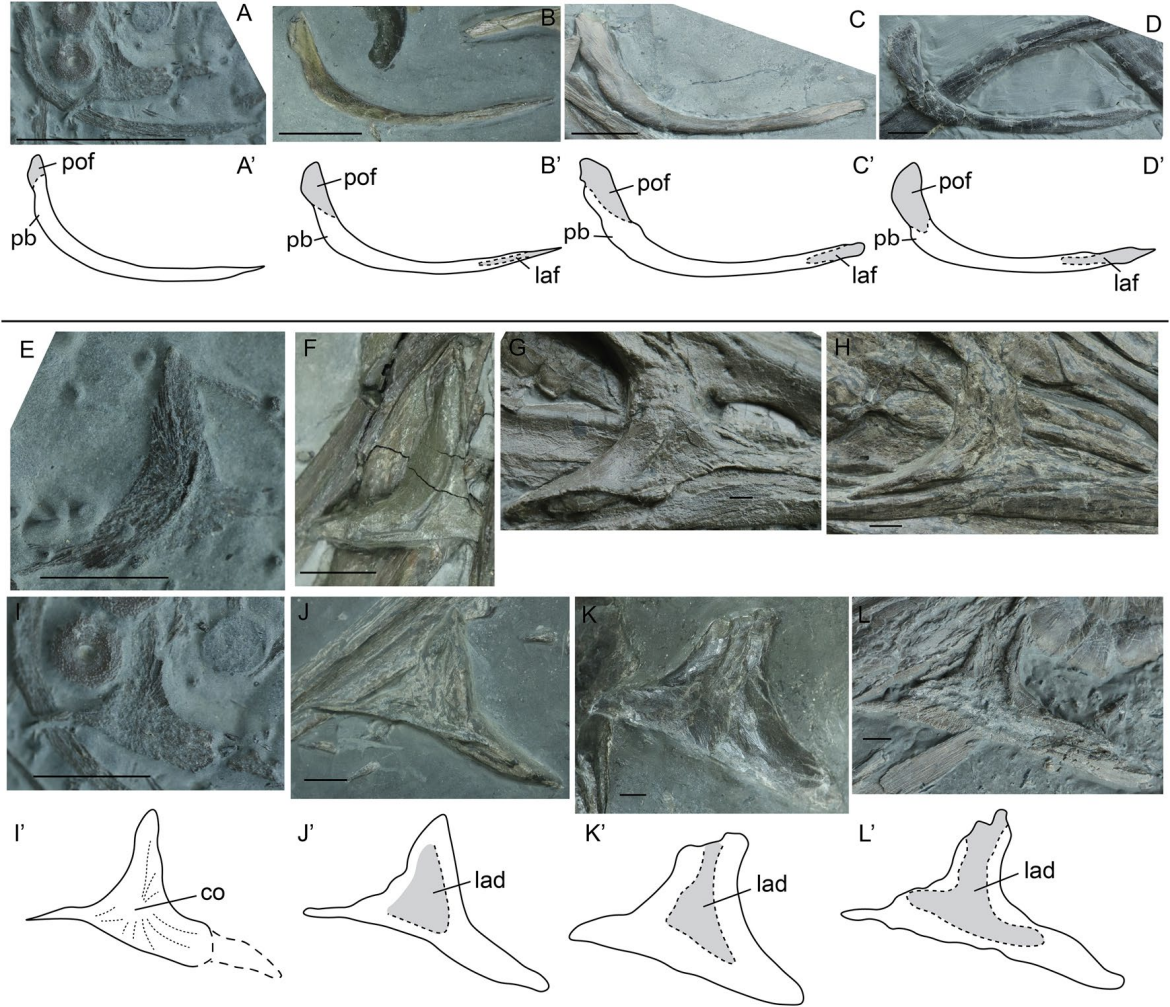
Jugals in lateral view (**A**–**D**) and lacrimals in lateral view (**E**–**H**) and medial view (**I**–**L**) of selected specimens of *Stenopterygius.* Interpretative drawings denoted by an apostrophe with their corresponding letter. (**A**) stage 3 embryo *S. quadriscissus* SMNS 80234; (**B**) postnatal 1 SMNS 81806 *Stenopterygius* sp*.* (**C**) postnatal 2 *S. quadriscissus* SMNS 81958; (**D**) sexually mature *S. quadriscissus* SMNS 80234 (**E**) stage 3 embryo *S. quadriscissus* SMNS 80234; (**F**) postnatal 1 *S. quadriscissus* SMNS 51139; (**G**) sexually mature *S. triscissus* SMNS 96899; (**H**) sexually mature *S. quadriscissus* SMNS 51948 (**I**) stage 3 embryo *S. quadriscissus* SMNS 80234; (**J**) postnatal 2 *S. quadriscissus* SMNS 51551; (**K**) sexually mature *S. quadriscissus* SMNS 50376; (**L**) sexually mature *S. quadriscissus* SMNS 81961. *co* centre of ossification, *lad* lacrimal medial depression, *laf* lacrimal facet, *pb* posterior bulge, *pof* postorbital facet. Scale bar: (**A–L**) = 20 mm.

The posterolateral margin of the lacrimal is thicker than the anterolateral edge, and contributes to the anterior orbital rim. Medially, the lacrimal has a distinct depression (Fig. 2I–K), which is most apparent in the dorsal and central regions. This depression possibly housed (the posterior part of) the salt glands^33,34^. The lacrimal articulates with the prefrontal, the maxilla, and the jugal via scarf joints and the premaxilla and the nasal in butt joints. The latter two contacts are not visible in all articulated specimens due to taphonomic distortion and intraspecific variability.

The lacrimal is well ossified by embryonic stage 3, with a centrally positioned ossification centre (Fig. 2E–I). From stage 3 onward a distinct ridge is present on the lateral side of the element indicating the anteriormost part of the circumorbital area (Fig. 2E–H). This ridge is still pronounced in postnatal stage 1, but is less pronounced in sexually mature specimens (Fig. 2F–H). The dorsal process, which interdigitates with the prefrontal, widens over ontogeny, giving the lacrimal a more robust morphology in the adult stage. The articulation with the prefrontal is therefore likewise enlarged over ontogeny. The lateroventral surface of the lacrimal, which accommodates the maxilla and jugal facets, is straight in the embryonic and juvenile phases (apart from one stage 4 embryo of *S. quadriscissus*: MHH 1981/33, not figured). However, in the sexually mature stage the surface is often irregular; the most extreme case being *S. triscissus* SMNS 96899 (Fig. 2G). There is however no evidence for interspecific variation in this morphology. The medial surface of the lacrimal is less ossified than the lateral surface in embryonic stage 3 (Fig. 2E–I). The medial depression is only apparent in the postnatal 2 and sexually mature stages, although its presence cannot be assessed in postnatal stage 1. The distinct edges of the orbital rim and external naris are apparent in lateral view in all stages. In the sexually mature stage, these edges are also visible in medial view, seemingly projecting more medially over ontogeny and thereby thickening the borders of the medial depression (Fig. 2L).

##### Jugal

The jugal is well ossified by embryonic stage 3. It is curved parabolically in the embryonic and postnatal 1 stages, but the more anterior portion straightens gradually over ontogeny, acquiring a more angular shape in the sexually mature stage (Fig. 2A–D). There is a bulge on the posterior end of the element ventral to the postorbital facet. The bulge emerges in a more dorsal position on the jugal in embryonic stage 3 but gradually shifts ventrally over ontogeny as the jugal becomes more angular (Fig. 2A–D). The postorbital facet is clearly visible in all ontogenetic stages, whereas the maxillary facet is first apparent from postnatal stage 1 onwards (Fig. 2B–D). The lacrimal facet lengthens relative to the entire jugal over ontogeny, and its groove deepens.

##### Prefrontal

The prefrontal articulates in scarf- or step-joints with the lacrimal, nasal, postfrontal, and frontal.

The prefrontal is well ossified early in ontogeny. In stage 3 embryos onward, it becomes progressively more quadrangular in overall shape. The orbital roof is very prominent and better ossified than the rest of the element in embryonic stages 3 and 4, but loses prominence relative to the postfrontal facet and medial margin over ontogeny. The nasal facet is not visible in the isolated embryonic elements of stage 3; however, this may be a preservational artefact. The nasal facet is less strongly textured than the lacrimal and postfrontal facets from stage 4 onwards, indicating differences in suture morphology (Supplemental Fig. 5A–E).

##### Postorbital

The postorbital articulates in scarf- or step joints with the jugal, quadratojugal, squamosal and postfrontal.

The postorbital is well ossified in embryonic stage 1 (Fig. 1). The circumorbital area is present in all observed ontogenetic stages, but is most prominent in the later stages. The shape of the postorbital changes over ontogeny from straighter to slightly more curved, with the squamosal and postfrontal facets shifting dorsally towards its dorsal-most point. The ventral portion of the postorbital, containing the jugal and quadratojugal facets, broadens antero-posteriorly over ontogeny (Supplemental Fig. 6A–D).

##### Postfrontal

Posteriorly, the postfrontal forms a complex overlapping suture with the lateral supratemporal and partially overlaps the postorbital; anterolaterally and also medially it overlaps the prefrontal.

The postfrontal is already well ossified in embryonic stage 1 (Fig. 1), with the best ossified areas in dorsal view being the supraorbital flange and medial edge forming the lateral portion of the upper temporal fenestra (UTF) (Fig. 1). The medial plate, containing the prefrontal contact, is less ossified. This persists into embryonic stage 3 (Supplemental Fig. 6E,H). In ventral view, the supratemporal facet is already visible in embryonic stage 3, but the frontal facet can only be distinguished from embryonic stage 4 onwards (Supplemental Fig. 6E–G). The shape and proportions of the postfrontal remain similar throughout ontogeny, although the supraorbital flange is less prominently visible in dorsal view in the postnatal 2 and sexually mature stages than in embryonic stage 3 (Supplemental Fig. 6H–J). This is due to ossification differences, rather than changes in relative contribution to the orbital roof.

#### Cheek area

##### Quadratojugal

The quadratojugal has distinct lateral facets for articulation with the postorbital and the squamosal, and is often obscured in articulated specimens. On the medial surface, a socket-like articular facet for the quadrate is visible posteroventrally.

The quadratojugal undergoes a shape change from embryonic stage 3 onwards. In embryonic stage 3, it is triangular in lateral and medial view, and the quadrate process and quadrate facet are already apparent and this region is the most well ossified part of the element. The ossification centre of the quadratojugal is situated around the quadrate facet (Supplemental Fig. 7A,E). The lateral portions forming the not-yet-distinct postorbital and squamosal facets are less well ossified. No isolated quadratojugals were found in stage 4 embryos. From postnatal 1 stage onward, the quadratojugal becomes more quadrangular in shape. It is very slender in the postnatal 1 and 2 stages, but becomes more robust in sexually mature individuals (Supplemental Fig. 7A–D). The postorbital facet has a distinctive border and texture in postnatal stage 2, but these are less clear in sexually mature specimens (although this may be partially attributed to interspecific variation: the sexually mature specimen is referred to *S. triscissus*). The squamosal facet is slender and smooth in all postnatal stages. The anteroventral- and posterodorsal extensions of the quadratojugal become proportionately smaller relative to the rest of the element over ontogeny. This is best seen in medial view, although the anteroventral flange is partially broken in specimens SMNS 50376 and SMNS 50815 (Supplemental Fig. 7D,G). In quadratojugals of postnatal specimens preserved in medial view, the dorsal margin displays two distinct bulges, which are not as apparent in lateral view (compare Supplemental Fig. 7C–D,F–H). The indention between the bulges corresponds to the location of the lateral squamosal facet. It is possible that this area is less ossified and collapses more easily than the surrounding bone, especially when exposed in medial view. Aggressive preparation would exaggerate this apparent morphology, which is certainly the case for SMNS 50376 (Supplemental Fig. 7G). There is little qualitative ontogenetic variation in the postnatal morphology of the medial surface of the quadratojugal; postnatal 1 specimen SMNS 51959 and sexually mature specimen SMNS 50376 have similar morphologies (Supplemental Fig. 7F–G). However, there is a large difference in width between sexually mature specimens SMNS 50376 and SMNS 80234, which suggests an unexplained source of variation (Supplemental Fig. 7G–H).

##### Squamosal

**Figure 3:**
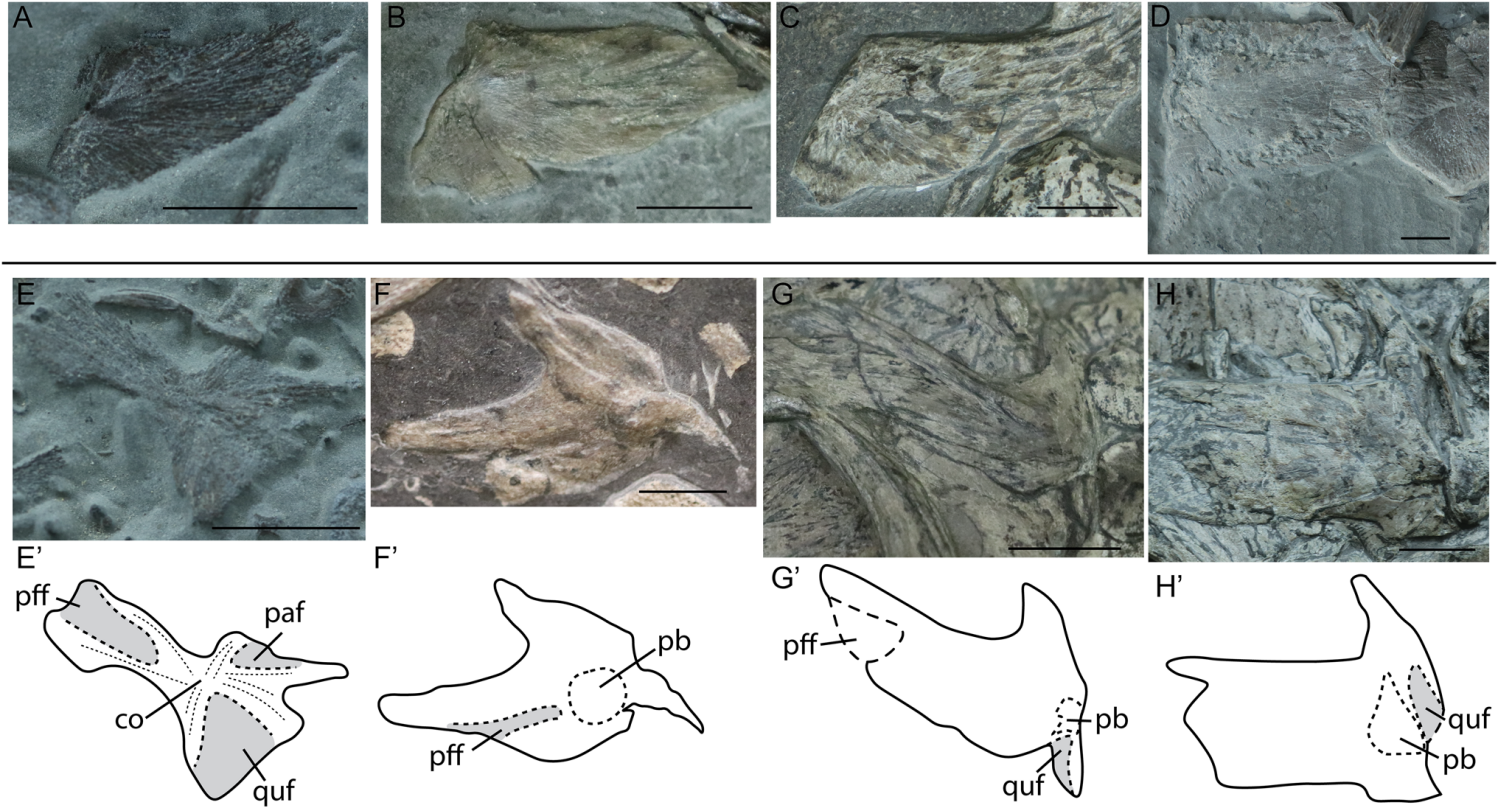
Squamosals in medial? (**A**) and lateral (**B**–**D**) views and supratemporals in posterodorsal (**E**–**H**) view of selected specimens of *Stenopterygius*. Interpretative drawings denoted by an apostrophe with their corresponding letter. (**A**) stage 3 embryo *S. quadriscissus* SMNS 80234; (**B**) postnatal 1 *S. quadriscissus* SMNS 51139; (**C**) postnatal 2 *S. quadriscissus* SMNS 50003; (**D**) sexually mature *S. quadriscissus* SMNS 81961; (**E**) stage 3 embryo *S. quadriscissus* SMNS 80234; (**F**) stage 4 embryo *S. quadriscissus* SMNS 54062; (**G**) postnatal 1 *S. quadriscissus* SMNS 82046; (**H**) sexually mature *S. quadriscissus* SMNS 51554. *co* centre of ossification, *paf* parietal facet, *pb* posterior bulge, *pff* postfrontal facet, *quf* quadrate facet. Scale bar: (**A–H**) = 10 mm.

The squamosal partially covers the quadratojugal and postorbital. It is observed from at least stage 2 onwards and is already well ossified in embryonic stage 3 (Fig. 3A). The ossification centre lies posteromedially, along the edge forming the quadrate foramen (as seen in lateral view) (Fig. 3A). The shape of the squamosal is relatively conservative, remaining triangular with relatively similar proportions over ontogeny. However, the angle between the dorsal and posterior margin decreases from a wider, more obtuse angle in embryos and juveniles to a right angle in the sexually mature stage (Fig. 3A–D).

##### Supratemporal

The supratemporal is tripartite in dorsolateral view (Fig. 3E–H). It consists of an anterior, medial, and a ventral ramus; the latter contains the quadrate facet.

The supratemporal is observed in all stages and is already relatively well-ossified in embryonic stage 3, but much better ossification is observed perinatally (Fig. 3E,F). It ossifies from a central posterodorsal position and broadens over ontogeny. This central ossification centre continues as a posterior bulge in embryonic stage 4 and postnatal specimens. The facet areas become less prominent from embryonic stage 3 to the sexually mature stage and both the anterior and medial rami thicken dorsoventrally. Due to preservation, it is difficult to assess if there is a change in the medial ramus or the ventral extension of the ventral ramus.

#### Skull roof

##### Frontal

The frontal is extensively overlapped by the parietal, prefrontal and nasal, which limits the exposed surface when in articulation.

**Figure 4:**
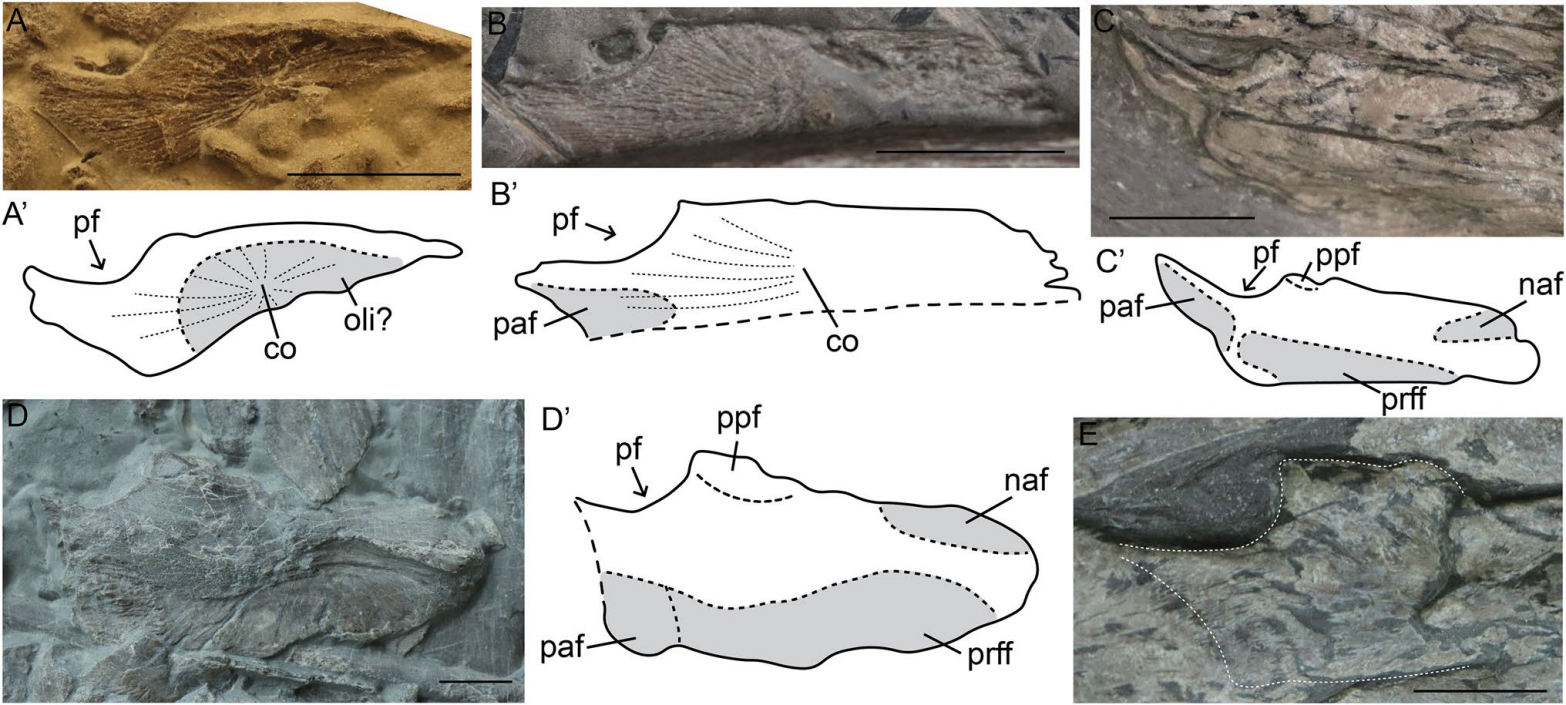
Frontals of selected specimens of *Stenopterygius* in ventral (**A**) and dorsal (**B**–**E**) view. Interpretative drawings denoted by an apostrophe with their corresponding letter. (**A**) stage 3 embryo *S. quadriscissus* SMNS 80234; (**B**) stage 4 embryo *S. quadriscissus* SMNS 81961; (**C**) postnatal 1 *S. quadriscissus* SMNS 82046; (**D**) sexually mature *S. quadriscissus* SMNS 80234; (**E**) postnatal 2 *S. quadriscissus* SMNS 54026. *co* centre of ossification, *naf* nasal facet, *oli* olfactory lobe indentation, *paf* parietal facet, *pf* parietal foramen, *ppf* process anterior to the parietal foramen, *prff* prefrontal facet. Scale bar: (**A**–**E**) = 10 mm.

**Figure 5:**
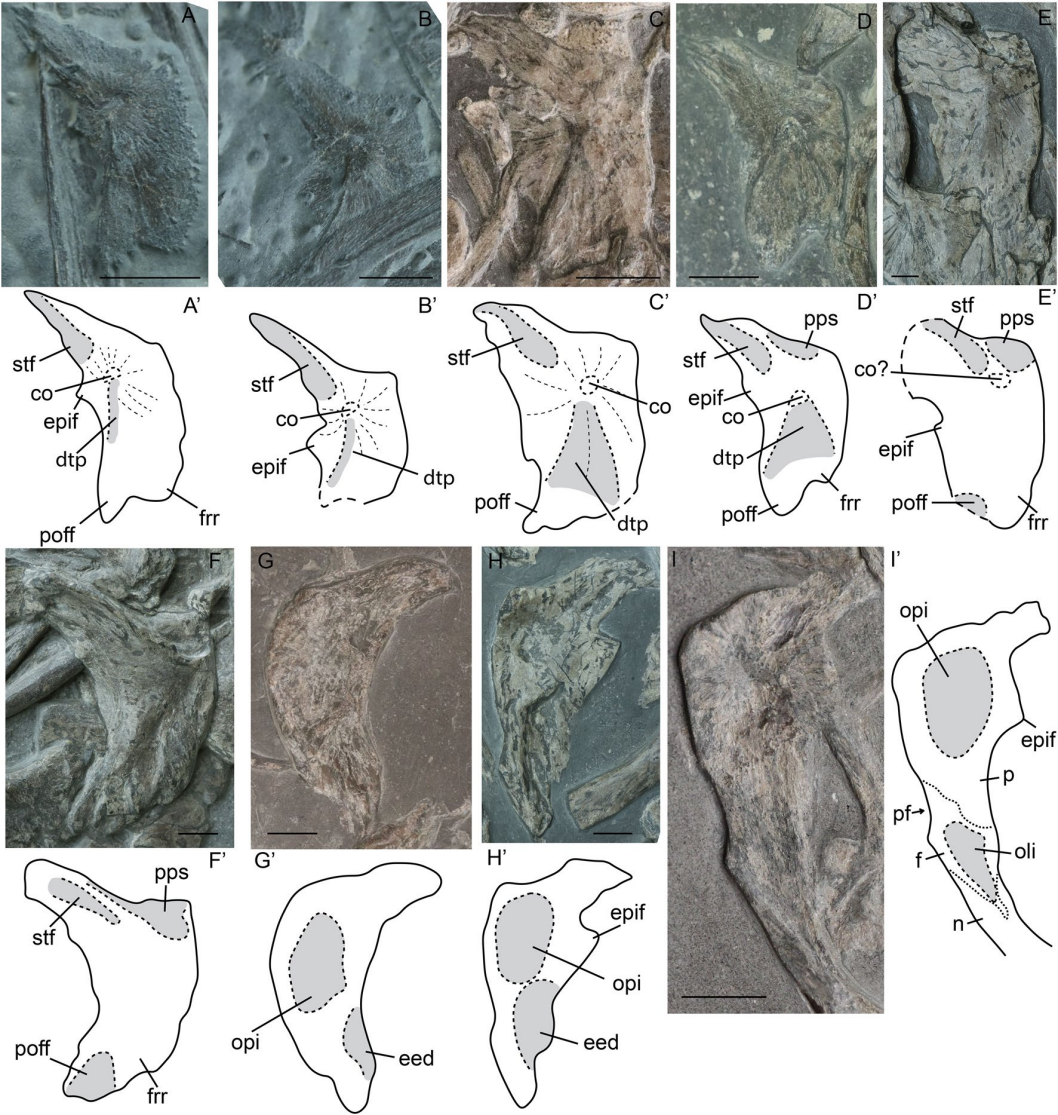
Parietals of selected specimens of *Stenopterygius* in dorsal (**A**–**F**) and ventral (**G**–**I**) view. Interpretative drawings denoted by an apostrophe with their corresponding letter. (**A**) stage 3a embryo *S. quadriscissus* SMNS 80234; (**B**) stage 3b embryo *S. quadriscissus* SMNS 80234; (**C**) stage 4 embryo *S. quadriscissus* SMNS 54062; (**D**) postnatal 1 *Stenopterygius* sp. SMNS 51140; (**E**) postnatal 2 *S. quadriscissus* SMNS 54026; (**F**) sexually mature *Stenopterygius* sp. SMNS 51843; (**G**) postnatal ?2 *S. quadriscissus* 50183; (**H**) postnatal 2 *S. quadriscissus* SMNS 51551; (**I**) stage 4 embryo *S. quadriscissus* SMNS 54064. *co* centre of ossification, *dtp* dorsal triangular process, *eed* extra-encephalic depression, *epif* epipterygoid facet, *f* frontal, *frr* frontal ramus, *n* nasal, *oli* olfactory indentation, *opi* optic lobe indentation, *p* parietal, *pf* parietal foramen, *poff* postfrontal ramus, *pps* parietal shelf, *stf* supratemporal facet. Scale bar: (**A**–**I**) = 10 mm.

Isolated embryonic frontals were identified in stages 3 and 4, with the stage 3 frontal preserved in ventral view and the stage 4 frontal in dorsal view (Fig. 4A,B). The frontal is already relatively well ossified in stage 3, especially the medial edge. Its ossification centre is directly above the olfactory lobe (Fig. 4A,B). In embryonic stage 4, the medial edge is straighter than in stage 3 (Fig. 4A,B). The facet for the overlapping parietal is poorly visible (Fig. 4B). Although the medial edge of the frontal is well ossified perinatally, the interfrontal suture is less ossified than the frontal-nasal or frontal-parietal sutures (Fig. 5I). A depression housing the olfactory lobe was observed in both embryonic stages (Fig. 4A, Fig. 5I). In postnatal stage 1, a medial process starts to form just anterior to the anterior border of the parietal foramen. This process increases in size over ontogeny (Fig. 4C–E), and forms an interdigitating suture with the contralateral frontal. The facets of the overlapping nasal, prefrontal and parietal become clear in postnatal stage 1 (Fig. 4C). Between the postnatal 1 and sexually mature stages there is an apparent shape change in the facets from more quadrangular and elongate to rounder and broader (Fig. 4C,D). It is possible the shape-change tracks minor positioning differences of bones over ontogeny, but this is equivocal given the small sample size.

##### Parietal

The parietal overlaps the frontal anteriorly. The dorsal surface of the supratemporal process of the parietal has a facet for articulation with the supratemporal. On the ventral surface, indentations which accommodated the optic lobe and extra-encephalic indentions are present.

Differences in ossification of the parietal exist among stage 3 embryos associated with SMNS 80234 (Fig. 5A,B), as previously noted for braincase elements^5^. The texture on the dorsal surface of the parietal is rougher and the medial edge is formed by bony spicules in stage 3a, whereas it is smooth in stage 3b. Likewise, in stage 3a the posteromedial margin is continuous with the supratemporal process, whereas in 3b it is mediolaterally oriented and offset from the supratemporal process, a state that persists into the postnatal stages. The ossification centre is located dorsal to the optic lobes as in other diapsids^35^. Radiation of bone fibres from this centre persists into postnatal stage 1, but becomes less prominent in postnatal stage 2 and is no longer visible in sexually mature individuals. Dorsally, a distinct triangular platform is present anterior to the ossification centre in embryonic stage 4 and postnatal stage 1 (Fig. 5C,D). Two elevated ridges delineating this platform originate from the ossification centre. These ridges decline in prominence toward the sexually mature stage as the dorsal surface of the parietal becomes more rounded medio-laterally.

There is a distinct difference in overall ossification between stage 3b and stage 4 embryos (Fig. 5B,C). Stage 4 embryos display a level of ossification similar to postnatal stage 1. The posterior parietal shelf is absent in all embryonic stages and underdeveloped to absent in postnatal stage 1; the shelf is present in postnatal stage 2 and sexually mature specimens. In the latter, the shelf is often anteriorly delineated at its dorsal margin from the rest of the parietal (Fig. 5A–F). Although the parietal is well ossified perinatally, the interparietal suture is not well developed (Fig. 5C,I). In many postnatal stage 1 and 2 specimens, the interparietal suture is likewise underdeveloped, whereas in sexually mature individuals, isolated parietals are rare. Moreover, the medial edge of the parietal undergoes several ontogenetic shape changes. It is anteroposteriorly straight in embryonic stages 3b and 4 (Fig. 5B,C), but becomes more rounded postnatally (Fig. 5D,E,G,H). In the sexually mature stage, only the anteromedial edge remains rounded, whereas the posteromedial edge—likely the location of the most tightly sutured portion of the interparietal suture—is again straight (Fig. 5F). The relatively small facet for the postfrontal is more apparent in the sexually mature and possibly postnatal 2 stages than in earlier stages. The lateral margin of the parietal, which forms the medial side of the upper temporal fenestra, is more curved in the sexually mature stage than in all other stages, indicating that the UTF may become wider over ontogeny.

Two parietals of postnatal 2? specimens as well as one stage 4 embryo of *S. quadriscissus* were preserved in ventral view (Fig. 5G–I). All three display a central circular indentation ventral to the ossification centre and the juveniles show a less pronounced anterolateral semilunate indentation, posterolateral to the parietal foramen. These housed the optic lobe and an extra-encephalic depression, respectively, as in, *Hauffiopteryx typicus*, *Ichthyosaurus communis, Nannopterygius enthekiodon* and *Platypterygius australis*^36–39^.

In dorsal view, there is a triangular process lateral to the ossification centre in embryonic and less prominently in postnatal stages. This is the epipterygoid facet, likely a ventrally directed process that becomes (partially) visible dorsally after flattening. An epipterygoid facet has been described in *Ichthyosaurus communis*, *Undorosaurus gorodischensis, Ophthalmosaurus icenicus* and *Platypterygius australis*^37,38,40,41^.

#### Palate

##### Vomer

The vomer is an elongated anteriorly tapering element. In medial view, it has a distinct groove as a facet for the anterolateral palatal ramus of the pterygoid. Anteroventral to this groove lies a flat surface: the antimeric vomer facet. Posterodorsally, there is a small crest at the posterior margin of the pterygoid groove. It has a rougher texture than the rest of the vomer, which continues posteroventrally into the palatine facet. Posteromedioventrally there is a small extra groove for the pterygoid, as in *Ophthalmosaurus icenicus*^41^. Dorsal to this groove is a roughened surface (Supplemental Fig. 8H). The vomer is usually obscured in articulated specimens.

Small sample size prevents detailed assessment of ontogenetic variation in the vomer. In embryonic stage 3, the vomer is poorly ossified. No distinct facets are visible at this stage. The vomer ossifies from a posteroventral centre, potentially near the internal narial opening (Supplemental Fig. 8E). In embryonic stage 4, it is substantially more ossified and the crest and roughened surface are becoming distinctly visible. The degree of ossification is higher anteriorly. Interestingly the degree of ossification is lower near the original centre of ossification, potentially related to increased vascularization around the internal naris (Supplemental Fig. 8G). In postnatal stage 1, the anterior pterygoid and vomer facets are visible. However, other facets are less prominent to virtually absent (likely partially due to preparation) (Supplemental Fig. 8F). The crests slope with a wide angle into the posterior roughened surface and posterior margin in sexually mature individuals; in embryos and postnatal stage 1 this slope approaches a right-angle.

##### Palatine

The palatine is an oval element on the lateral side of the central palate. It has a lateral and a medial process anteriorly, which contact the maxilla and vomer, respectively. In dorsal? view, a distinct ridge runs along the lateral edge of the palatine into the maxillary process (Supplemental Fig. 8A–D). We were unable to identify a palatine in any sexually mature specimens, as it is often obscured in articulated material.

In embryonic stage 3, the palatine is poorly ossified, but the distinct anterior processes are already present. Posteriorly the palatine is forked, which is not the case in postnatal stages. The palatine has its ossification centre posteriorly on the midline, just anterior to this posterior bifurcation (Supplemental Fig. 8A). Morphology seems relatively conserved in the postnatal stages.

##### Pterygoid

The triradiate quadrate ramus of the pterygoid sensu^41^ appears flat in ventral view, with the dorsal flange sometimes invisible, whereas in dorsal view its triradiate structure is easily observed.

**Figure 6:**
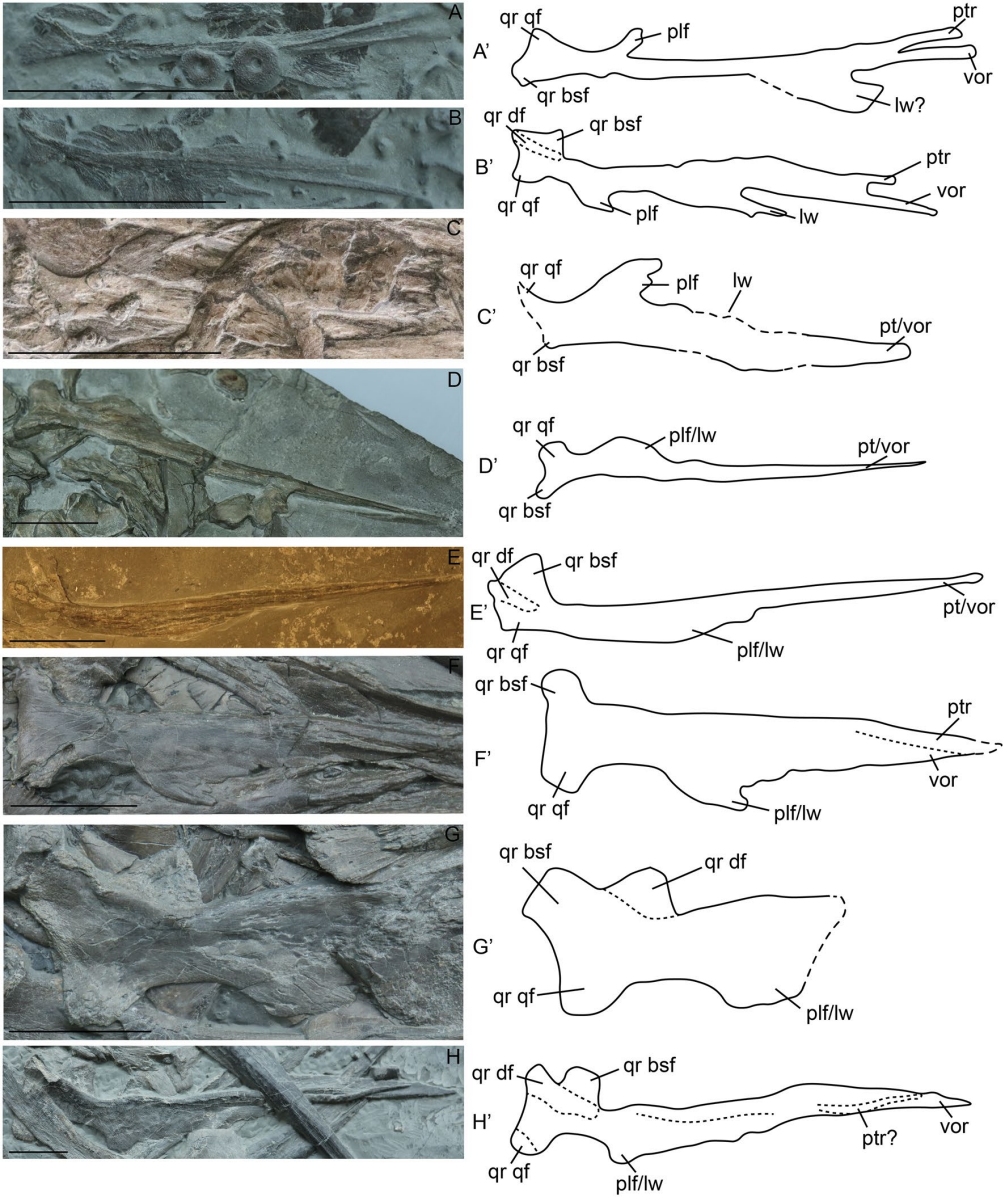
Pterygoids of selected specimens of *Stenopterygius* in ventral (**A**,**C**–**D**,**F**–**G**) and dorsal (**B**,**E**,**H**) view. Interpretative drawings denoted by an apostrophe with their corresponding letter. (**A**) stage 3 embryo *S. quadriscissus* SMNS 80234; (**B**) stage 3 embryo *S. quadriscissus* SMNS 80234; (**C**) stage 4 embryo *S. quadriscissus* SMNS 54062; (**D**) postnatal 1 *S. quadriscissus* SMNS 51139; (**E**) postnatal ?2 *Stenopterygius quadriscissus* 50183; (**F**) postnatal 2 *S. quadriscissus* SMNS 81958; (**G**) sexually mature *S. quadriscissus* SMNS 81961; (**H**) sexually mature *S. quadriscissus* SMNS 80234. *lw* lateral wing, *plf* palatine facet, *ptr* antimeric pterygoid ramus, *qr bsf* quadrate ramus, medial (basisphenoid) flange, *qr df* quadrate ramus dorsal flange, *qr qf* quadrate ramus quadrate flange, *vor* vomerine ramus. Scale bar (**A**–**H**) = 30 mm.

The pterygoid is poorly ossified in embryonic stage 3, and the anterior margin of the palatal ramus is forked (Fig. 6A,B). In embryonic stage 4, the pterygoid is better ossified and the anterior end is not bifurcated (Fig. 6C), a state which persists in juveniles. However, the bifurcation reappears in sexually mature specimen SMNS 80234 as the thicker medial process is found turned separately from the lateral wing (Fig. 6H). We are unsure whether this represents normal morphology given the small sample size. In the embryonic stages, the lateral part of the palatal ramus develops separately from the palatine facet, whereas they are continuous from postnatal stage 1 onwards (Fig. 6A–H). The quadrate ramus is triradiate throughout ontogeny. The dorsal flange is less prominent than the medial and lateral flanges prior to postnatal stage 2, but all flanges are similar in size in sexually mature individuals. The lateral and medial flanges appear flat and quadrangular in ventral view in embryonic stage 3, but bulge in postnatal stages. The size of the quadrate ramus relative to the length of the pterygoid remains relatively constant over ontogeny, as does the degree of convexity and the relative distance between the edge of the quadrate ramus and the edge of the palatal flange. This suggests that there is relatively little change in the shape and relative size of the subtemporal fenestra.

#### Lower jaw

##### Dentary

By embryonic stage 2, the dentary is well-ossified and dentigerous, although the teeth are very loosely set in the alveolar groove in this stage. The dentary fossa is vaguely present in stage 3 and 4 embryos, more so in postnatal stage 1, and is clearly defined from postnatal stage 2 onwards (Supplemental Fig. 9E–H).

##### Splenial

The splenial is an elongated element which tapers slightly in its posterior portion and forms the majority of the medial side of the lower jaw. Its anterior end has dorsal and ventral processes which form the posterior mandibular symphysis. The dorsal process is about one-third of the length of the ventral one. The splenial is already well-ossified in embryonic stage 3 (Supplemental Fig. 9A). In postnatal stage 1, it is fully ossified with a smooth surface texture (Supplemental Fig. 9B). From postnatal stage 2 onwards, the medial surfaces of the dorsal and posterior ventral process are roughened. Moreover, the ventral process displays a distinct facet for the contralateral splenial. The roughened surface and ventral facet indicate a tight symphyseal articulation in these stages (Supplemental Fig. 9C,D). However, the symphyseal facets are less apparent in postnatal stage 1 and are absent in embryonic stage 3 (no splenials were preserved in medial view in stage 4 embryos). The relative length of the dorsal and ventral processes is consistent throughout ontogeny.

##### Surangular

**Figure 7:**
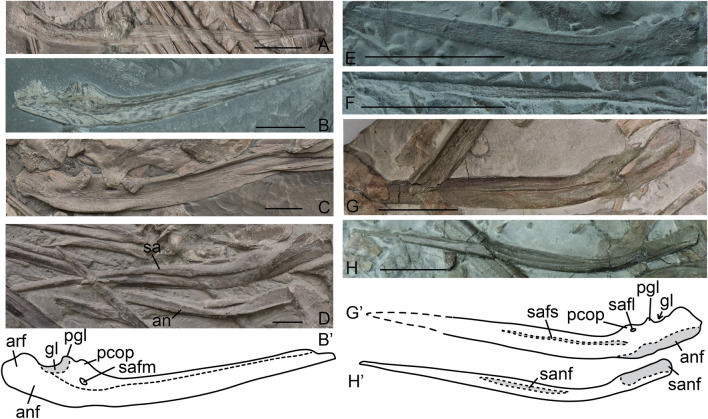
Surangulars in medial (**A**–**C**) and lateral (**D**,**E**,**G**) and angulars in medial (**D**,**F**,**H**) view of selected specimens of *Stenopterygius*. Interpretative drawings denoted by an apostrophe with their corresponding letter. (**A**) stage 4 embryo *S. quadriscissus* SMNS 81961; (**B**) postnatal 2 *S. quadriscissus* SMNS 51551; (**C**) sexually mature *S. quadriscissus* SMNS 81961; (**D**) sexually mature *S. quadriscissus* SMNS 80234; (**E**) stage 3 embryo *S. quadriscissus* SMNS 80234; (**F**) stage 3 embryo *S. quadriscissus* SMNS 80234; (**G**) postnatal 1 *S. quadriscissus* SMNS 51139; (**H**) postnatal 1 *S. quadriscissus* SMNS 51139. *anf* angular facet, *arf* articular facet, *gl* glenoid, *pcop* paracoronoid process, *pgl* preglenoid process (insertion MAME), *safl* lateral surangular foramen, *safm* medial surangular foramen, *safs* surangular fossa, *sanf* surangular facet. Scale bar: (**A**–**H**) = 30 mm.

The surangular is clearly visible in embryonic stage 2 and is well ossified relative to braincase and skull roof elements in embryonic stage 3 (Fig. 1). The surangular is close to fully ossified perinatally and fully ossified in postnatal stage 1 (Fig. 7A,G). The surangular fossa is visible in embryonic stage 3, but the lateral surangular foramen only appears postnatally (Fig. 7E,G). Medially, the glenoid fossa is well defined in the postnatal stages as a concave surface, which is roughened and bordered by a prominent ridge anteriorly (Fig. 7B,C). This ridge is interpreted as the insertion point for the external mandibular adductor muscle (MAME). The glenoid fossa is not defined in perinatal embryos, however the ridge for the insertion of MAME is visible in embryonic stages 3 and 4 (Fig. 7A,E; preglenoid process sensu^42^). The paracoronoid process is apparent in sexually mature individuals as a bulge just anteriorly to the preglenoid process. In sexually mature specimens and embryonic stages 3 and 4, the paracoronoid process descends slightly towards the preglenoid process; however, in juveniles the paracoronoid process does not descend towards the preglenoid process, but the two contact directly (Fig. 7B). This suggests a phase of bone remodeling in or around the paracoronoid process of the surangular in *S. quadriscissus* between postnatal stages 1 and 2. A foramen is present on the medial surface of the surangular ventral to the paracoronoid process in all postnatal stages. We found no evidence of an ossified coronoid bone at any stage.

##### Angular

Posteromedially, the angular has a quadrangular facet for the surangular. The surangular facet is a depression bordered by distinct margins ventrally and anteriorly (Fig. 7D,F,H).

We could not assess whether the anterior extent of the lateral exposure of the surangular changes over ontogeny due to taphonomic displacement and distortion. The degree of posterior exposure of the angular seems to be consistent over ontogeny. The angular is present in embryonic stage 2, relatively well-ossified by stage 3, and fully ossified from postnatal stage 1 onwards (Fig. 7D,F,H). The morphology of the surangular facet is consistent across all stages. In embryonic stage 3, the surangular facet is already prominently visible and the ventral margin is likewise defined; the anterior margin of the angular is however less developed (Fig. 7F).

##### Prearticular

The prearticular forms part of the posteromedial surface of the lower jaw. It contacts the surangular laterally, the articular posterolaterally, the angular ventromedially and the splenial anteromedially. The prearticular is an elongated element, which is deepest at its midpoint, forming a dorsal projection, and tapers anteriorly and posteriorly. Anteromedially, the prearticular has a V-shaped facet for the splenial and postero-ventromedially an elongated facet for the angular (Supplemental Fig. 10F–J). This morphology is fairly consistent over ontogeny, although the medial bulge is least apparent in the sexually mature stage (Supplemental Fig. 10J). Like the other dermal lower jaw elements, the prearticular is fairly well ossified at embryonic stage 3 and fully ossified by postnatal stage 1. Its ossification centre is in a central position just posterior to the splenial facet. The facets on the prearticular, especially the splenial facet, are already easily discernable by embryonic stages 3 and 4. In stage 3, there is a clear difference in ossification between the splenial facet and the rest of the element. In stage 4, the prearticular is ossified evenly throughout, but the original ossification lines clearly indicate the V-shaped facet. Ossification lines disappear postnatally. In some postnatal specimens (e.g., SMNS 50003, SMNS 51139), the boundaries of the facets are marked by ridges, with the ridge for the angular facet being the most prominent. In sexually mature specimens only the angular ridge remains distinct.

#### Splanchnocranium

##### Articular

The articular is a rounded element positioned on the posteromedial side of the lower jaw and contributing to the posterior margin of the glenoid, forming a distinct facet for articulation with the quadrate condyle. The articular is well ossified in all postnatal stages, but not well-ossified in embryonic stages 3 and 4 (Supplemental Fig. 10A–E). The glenoid facet is marked by a ridge in postnatal specimens, but is not seen in embryonic material. In postnatal specimens, the medial side of the articular displays a large convex surface and a distinct increase in depth posteroventrally, forming the prearticular facet (Supplemental Fig. 10C–E). In one sexually mature individual (SMNS 80234), the convex medial surface is divided into two halves. This seems to be a genuine feature, not altered by deformation, but is not seen in other sexually mature specimens, in which the morphology is akin to that of postnatal stage 2.

##### Hyoid apparatus

The paired first ceratobranchials (CB1) are slender and slightly curved, and are usually the only elements visible in ventral view. However, at least one sexually mature *S. quadriscissus* (SMNS 50165), and one postnatal 2? specimen of *S. triscissus* (SMNS 58276) also preserve an ossified hyoid corpus (Supplemental Fig. 11C,E). The hyoid corpus is a round element with flattened lateral edges, similar to that of *Hauffiopteryx typicus*^43,44^. Moreover, we tentatively identify the presence of the hyoid corpus in a stage 4 embryo, indicating that the element is fully ossified perinatally (Supplemental Fig. 11F). In the stage 4 embryo, the hyoid corpus bulges centrally and is very flat around the edges, something not observed in postnatal stages. This could be due to the ossification pattern of the element. The presence of ossified hyoid corpora in *Stenopterygius* is unexpected, but this element can be easily taphonomically lost due its size and loose connection to other elements. Moreover, it is easily overlooked as it does not have an overly characteristic shape. It is possible that an ossified hyoid corpus is more widely distributed within ichthyosauromorphs than previously reported.

The CB1 are observed from embryonic stage 3 onwards, with little morphological change over ontogeny (Supplemental Fig. 11A–E). In the sexually mature specimen SMNS 50165, the ceratobranchials are in contact with the hyoid corpus, whereas this is not the case in postnatal? 2 specimen SMNS 58276. It is not clear whether the connection in the sexually mature stage is due to increased ossification because the specimen is slightly overprepared. Based on the juvenile specimen, the connection between the ceratobranchials and hyoid corpus was largely cartilaginous.

##### Epipterygoid

Two sexually mature specimens of *S. quadriscissus* contain an ossified epipterygoid (Supplemental Fig. 11G–H). The epipterygoid is a dorsoventrally elongated element, containing a ventral footplate and a bulge in its mid-section (Supplemental Fig. 11H). The footplate, which would have contacted the pterygoid, is projected posteriorly. In vivo, the epipterygoid would have been angled slightly posteriorly (Supplemental Fig. 11G). The medial bulge is projected ventromedially and likely contacted a cartilaginous or ossified part of the prootic. Dorsally the epipterygoid ends in a narrow rod, which contacts the parietal.

An ossified epipterygoid is often observed in basal ichthyosaurians, such as *Chaohusaurus brevifemoralis* and *Besanosaurus leptorhynchus*, as well as in *Ichthyosaurus communis*^37,42,45,46^. As it is often obscured in slab mounted specimens, it is difficult to discern how variable the ossification of the epipterygoid is in *Stenopterygius*. As we did not identify any epipterygoids in immature stages it is possible that the variation is partially ontogenetic, but we are hesitant to state this with certainty.

### Size-independent identification of postnatal ontogenetic stages

We recognize the importance of well-defined reference stages for the assessment of skeletal maturity and propose three postnatal stages based on size and cranial morphology. However, there is some overlap between postnatal 1 and 2 around 300 mm mandible length and around 400 mm length between postnatal 2 and sexually mature individuals (Table S1; character states explained in Supplementary file S2).

The most important morphological features differing between the postnatal stages are as follows: *Dermatocranium*: **lacrimal**—walls of the medial depression underdeveloped in postnatal stages 1 and 2, well developed in sexually mature individuals; **jugal**—more lunate/parabolic + posterior bulge positioned on the dorsal ramus in postnatal 1 specimens, very angular jugal, posterior bulge in line with the horizontal ramus in sexually mature individuals, postnatal stage 2 intermediate; **squamosal**—posterior and dorsal edges form obtuse angle in postnatal stages 1 and 2 and form more of a right-angle in sexually mature specimens; **frontal**—process anterior to the parietal foramen underdeveloped in postnatal stage 1, well developed in the sexually mature stage, postnatal stage 2 intermediate; **frontal**—interfrontal connection weak in postnatal stages 1 and 2, well-connected or fused in sexually mature specimens; **parietal**—dorsal triangular platform present in postnatal stage 1, vaguely present in postnatal stage 2 and absent in the sexually mature stage; **parietal**—posterior parietal shelf absent in postnatal stage 1, present but not anteriorly delineated in postnatal stage 2, well developed and delineated in sexually mature individuals; **pterygoid**—lateral wing directed more anteriorly and sometimes not confluent with the anterior ramus in postnatal 1 and 2 specimens, directed more laterally in the sexually mature stage; **surangular**—preglenoid and paracoronoid process close to confluent in postnatal stage 2, clearly separate in other stages.

*Chondrocranium*: **basioccipital** condyle roughened compared to the extracondylar area in postnatal stage 1; extracondylar area and condyle smooth in postnatal 2 and sexually mature individuals^5^; **basioccipital** peg slender and elongated in postnatal stages 1 and 2, thick and broadened in sexually mature individuals^5^; **supraoccipital**—exoccipital facets directed ventrally in postnatal stages 1 and 2, often directed more lateroventrally in sexually mature specimens^5^.

### Postnatal suture closure and cranial fusion

All cranial elements are in contact by postnatal stage 1. Scarf and step joints involving the postorbital-squamosal-supratemporal, postorbital-jugal, prefrontal-postfrontal, and the entire lateral lower jaw are often fused in this stage. The posterior nasal joints as well as all joints involving the maxilla remain open and always display a distinct stepped suture line (Fig. 1). Among the suture closures, only the postorbital-jugal joint on rare occasion fuses with no discernable suture line. The butt joints along the midline of the cranium remain open in postnatal stages 1 and 2 (Fig. 1), but often fuse around sexual maturity. The connection of the posterior nasal joints and maxilla-jugal joint also appear tighter in sexually mature specimens, although fusion is rare, especially of the maxilla (Fig. 1).

## Discussion

### Phylogenetic implications

The phylogenetic position of Ichthyosauromorpha has not yet been resolved, although all recent analyses favour a diapsid or even sauropsid position^47–49^. Ingroup phylogeny is arguably better resolved with general consensus of the monophyly of the major clades, but little ingroup consensus^50–52^. This lack of consensus might be exacerbated by use of ontogenetically variable characters.

We found that characters pertaining to element-element contact are consistent over postnatal ontogeny in *Stenopterygius*, although caution should be taken regarding taphonomic and preparational alteration. However, we noted some commonly used phylogenetic characters show ontogenetic variation. For instance, all ontogenetic stages are characterized by a “lunate or j-shaped” jugal^53^, however there is a clear shape change from lunate to j-shaped over ontogeny affecting scoring of ref.^53^ character 31. Subsequent analyses should acknowledge ontogenetic variability of this character in *Stenopterygius* and potentially other closely related ichthyosaurs. Other circumorbital elements seem less affected by ontogeny; however, the lunate postorbital of *S. quadriscissus* shows a very small process for the squamosal, which is not visible in articulated material. This facet may potentially affect scoring in other (likely more primitive) ichthyosaurs as these could have a triradiate shape in isolation, but appear lunate when articulated with the squamosal. Some sexually mature specimens of *S. quadriscissus* have a pronounced descending process on the posterior edge of the squamosal^54^; this process is not as apparent in the other postnatal and prenatal stages. This is however only visible in isolated squamosals, not in articulation. Two characters pertaining to the parietal are ontogenetically variable in *S. quadriscissus* – the relative size of the supratemporal process and the parietal shelf^52,55^. The supratemporal process becomes relatively shorter over prenatal ontogeny following the ossification of the parietal shelf; postnatally it lengthens again relatively to the main body of the parietal. The parietal shelf originates postnatally, and is only prominent late in ontogeny (postnatal 2 and sexually mature stages). It is plausible that the presence and prominence of the parietal shelf is ontogenetically variable in other ichthyosaurs, especially around the base of Ophthalmosauridae^39,41^. Few phylogenetic characters pertain to the palatal elements, but one that might be confused due to ontogenetic change in *S. quadriscissus* is the direction of projection of the lateral wing of the palatal ramus of the pterygoid. The lateral wing is clearly projected anterolaterally in all immature stages, but the projection can be defined as lateral (or even posterolateral) in some sexually mature specimens. Moreover, the lateral wing in immature stages is often not confluent with the rest of the anterior ramus of the pterygoid and displays what is defined as a postpalatine process. The postpalatine process prevents the palatine from contributing to the subtemporal fenestra and is often used as a character^51^. Concerning the morphology of the lower jaw, the posterodorsal side of the surangular has been used as a source of phylogenetic characters, specifically the presence and prominence of the “coronoid process”^52^. However, definitions of the character vary. Originally it denoted the process of the surangular in the position of the absent coronoid (absence of a coronoid bone being either very common or equivocal in ichthyosaurs)^56^. Later it was reworked into only describing the morphology of a coronoid (if present)^52^. “Coronoid process” is in our opinion too vague: in euichthyosaurs, there are two processes to which the term coronoid process can refer: the preglenoid process and the paracoronoid process^41,42^. The paracoronoid process (if present) is the bulge on the surangular anterior to the glenoid; the process is located near and possibly homologous to the original coronoid process^42^. The preglenoid process is the pointed process anteriorly bordering the glenoid, which serves as the ventral attachment of the MAME (external mandibular adductor). In *S. quadriscissus*, both the preglenoid and paracoronoid processes are present throughout ontogeny. However, while they are clearly separated perinatally and in the sexually mature stage, they are close to confluent in postnatal stage 2. Confluence of the two processes should therefore not be used as a character in future analyses due to this complicated ontogenetic trajectory. Confluence of the two processes is observed in adult *Platypterygius australis*^38^, but the presence of a distinct preglenoid process is possibly ontogenetically variable in *Acamptonectes densus*^57^.

### Comparison of other ichthyosaur embryos to *Stenopterygius*

Fossil embryos are well documented in Ichthyosauriformes^14–17,22,32,58–61^. However, cranial material has been described or depicted for only a few, most notably *Platypterygius australis*^32^. The state of ossification of that embryo is very comparable to stage 4 *Stenopterygius*, with a well ossified dermatocranium, including the skull roof, and a less ossified, more rugose chondrocranium^32^. The palatal elements were slightly less ossified at the edges of the bone compared to the rest of the dermatocranium, which is comparable to the delay in ossification of the dermal palate in *Stenopterygius*^32^. Two other taxa with well depicted, albeit less thoroughly discussed, embryonic crania are *Chaohusaurus brevifemoralis* and *Leptonectes* cf*. tenuirostris*^15,17^. In both cases, the dermatocranium is visible and has a comparable degree of ossification to stage 4 *Stenopterygius*. An embryo of *Ichthyosaurus somersetensis* has been reported that is clearly in an early stage of ossification, possibly comparable to stage 1 or 2 *Stenopterygius*^18^. However, its anatomy has not been well-described.

### Biological implications of perinatal morphology

Overall, the cranium is highly ossified at birth in *Stenopterygius quadriscissus*, presumably related to the need to immediately locomote and feed independently. The appendicular regions also show a high degree of ossification, with the exception of more distal phalanges, and delayed ossification of the anterior periosteum in digit II^6,7^. There is therefore no osteological evidence that neonates required extensive parental care post-parturition. An absence of post-parturitional care is further supported by ontogenetic differences in diet^3^. Amongst other ichthyosaurian taxa, overall dermatocranial ossification is high in perinatal and neonatal individuals^15,17,32,58^.

**Figure 8:**
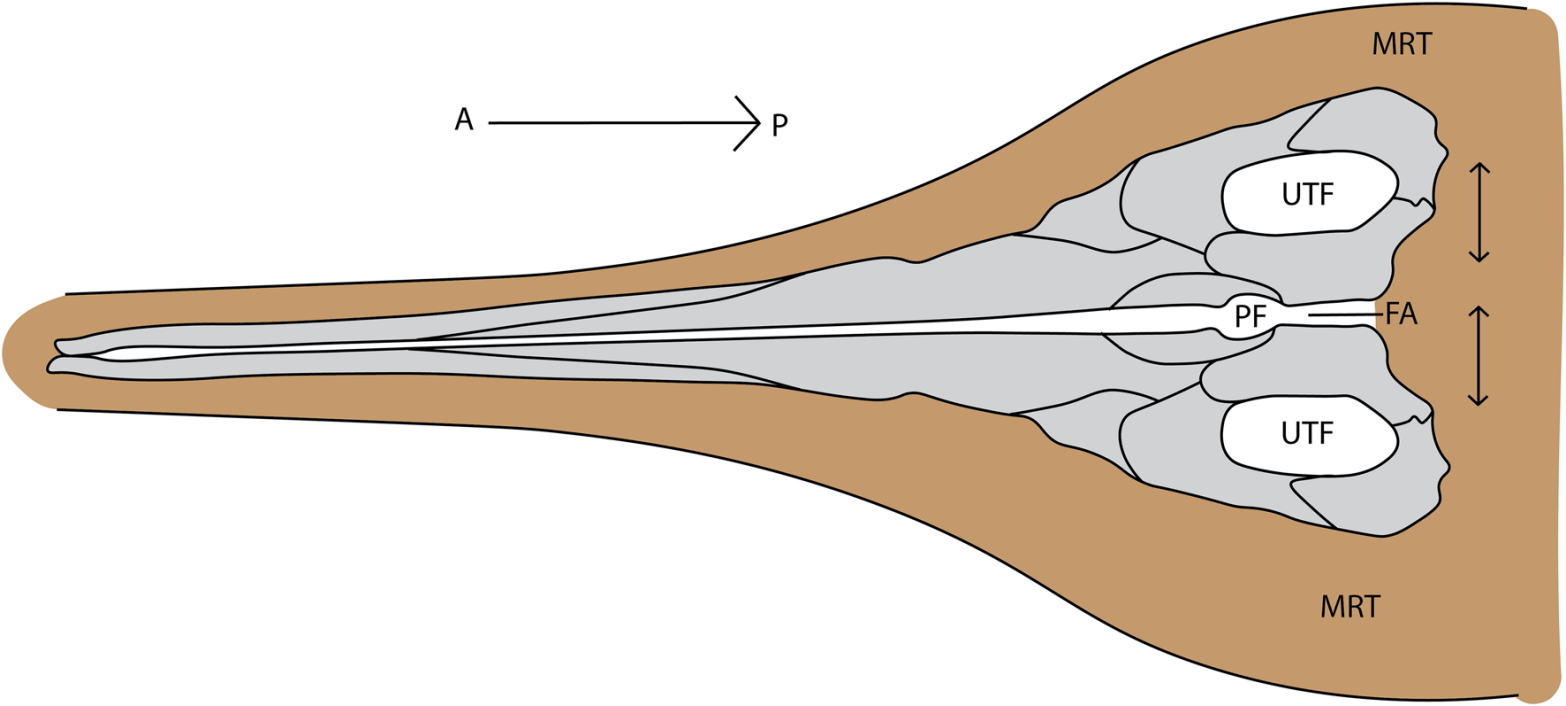
Schematic representation of the embryonic skull roof in the posterior reproductive tract. Embryonic skull is orientated in the most common position (tail-first birth), denoted by the arrow above the drawing (A = anterior, P = posterior). Fontanelle area is denoted in white. Arrows denote the possible movement of the two skull roof halves during birth contractions. *FA* fontanelle area, *MRT* maternal reproductive tract, *PF* parietal foramen, *UTF* upper temporal fenestra.

Despite high dermatocranial ossification at birth, ossification remains relatively weak near the cranial midline in *S. quadriscissus*. This is best demonstrated by an embryo associated with SMNS 54064, in which the parietal, frontal, and nasal remain in articulation in ventral view, with sutures completely closed (Figs. 1 and 5I). The bones are not broken along the midline, indicating that the joint with the contralateral bone is substantially weaker than the connection to bones on the same side of the skull. Moreover, on slab mounts, cranial roofs disarticulated at the midline, with the left or right side visible in lateral view and the contralateral element in dorsal view is quite common among juveniles (postnatal stages 1 and 2), but rare in sexually mature individuals. Midline sutures of the cranium are therefore still weak in most juveniles, but are established around sexual maturity and may even become obliterated in old individuals (e.g. *S. aaleniensis*—SMNS 90699^53^; *S. quadriscissus* SMNS 58881 (only the frontal)). This is best visible in the morphology of the frontal, in particular the process anterior to the parietal foramen. Midline sutures were also reported to be underdeveloped in the rostrum of a subadult specimen currently referred to *Muiscasaurus catheti*^62^. We hypothesize that weak midline ossification functions to prevent damage to the embryonic skull during birth, similar in function to the cranial fontanelles in mammals (Fig. 8). Fontanelles are not uncommon in oviparous sauropsids, whereby sometimes the frontal midline connection is tight at birth, whereas the parietal midline connection remains open^35,63–65^. Even though this morphology is not exclusive to ichthyosaurs^66,67^, the delayed midline closure of the frontal would therefore be an osteological adaptation to viviparity in the clade.

## Supplementary Information

Supplementary Information 1.

Supplementary Information 2.

Supplementary Legends.

Supplementary Table S1.

Supplementary Figure 1.

Supplementary Figure 2.

Supplementary Figure 3.

Supplementary Figure 4.

Supplementary Figure 5.

Supplementary Figure 6.

Supplementary Figure 7.

Supplementary Figure 8.

Supplementary Figure 9.

Supplementary Figure 10.

Supplementary Figure 11.
