# Supplementary material for: Ontogenetic variation in the skull of Stenopterygius quadriscissus with an emphasis on prenatal development
Source: Sci Rep. 2022 Feb 1;12:1707. doi: 10.1038/s41598-022-05540-0 (PMC8807662; doi:10.1038/s41598-022-05540-0)
Supplement: Supplementary file 1 — Supplementary Information 1. [file 41598_2022_5540_MOESM1_ESM.pdf]

Supplementary file 1: Parabasisphenoid

Miedema and Maxwell (2019) described the ontogenetic variation in the braincase of *Stenopterygius*, including the parabasisphenoid[1]. They included embryonic material known at the time of stages 3 and 4 (not naming it as such at the time of course). Other than 1 parabasisphenoid in a stage 1 embryo we did not find any other braincase elements in stages 1 and 2.
The parabasisphenoid in stage 1 is preserved in dorsal view very badly ossified compared to stage 3 (supplementary figure 1). As in stage 3 and 4 embryos the basioccipital facets are already apparent and divided in the midline. The parasphenoid is already fused to the basisphenoid in stage 1 which shows the two were fused closely after onset of ossification. Unfortunately, the region of the dorsum sellae is either not preserved or not ossified. In stage 3 the dorsum sellae has not closed anteriorly whereas it is fully closed in stage 4 (supplementary figure)[1]. On the whole the shape of the parabasisphenoid is more triangular in stage 1 than in the later stages. The triangular morphology is reminiscent of more basal ichthyosaurs such as *Chaohusaurus*, *Besanosaurus* and *Mixosaurus*[2,3,4].

For a full discussion of the parabasisphenoid in *Stenopterygius* see Miedema and Maxwell 2019[1].

1. Miedema, F. & Maxwell, E. E. Ontogeny of the braincase in Stenopterygius (Reptilia , Ichthyosauria) from the Lower Jurassic of Germany. *J. Vertebr. Paleontol.* **39**, (2019).
2. Bindellini, G., Wolniewicz, A. S., Miedema, F., Scheyer, T. M. & Sasso, C. D. Cranial anatomy of Besanosaurus leptorhynchus Dal Sasso & Pinna , 1996 (Reptilia: Ichthyosauria) from the Middle Triassic Besano Formation of Monte San Giorgio , Italy / Switzerland : taxonomic and palaeobiological implications. *PeerJ* **9**, e11179 (2021).
3. Huang, J. D. *et al.* The new ichthyosauriform Chaohusaurus brevifemoralis (Reptilia, Ichthyosauromorpha) from Majiashan, Chaohu, Anhui Province, China. *PeerJ* **7**, e7561 (2019).
4. Maisch, M. W. & Matzke, A. T. Observations on Triassic ichthyosaurs. Part I: Structure of the palate and mode of tooth implantation in Mixosaurus cornalianus (BASSANI, 1886). *Neues Jahrb. fur Geol. und Palaontologie - Monatshefte* **12**, 717–732 (1997).

Supplementary figure 1: parabasisphenoid

Parabasisphenoids in dorsal view of selected specimens of *Stenopterygius*. Interpretative drawings denoted by an apostrophe with their corresponding letter. **A**, stage 1 embryo *S. quadriscissus* SMNS 10460; **B**, stage 3 embryo *S. quadriscissus* SMNS 80234; **C**, stage 4 embryo *S. quadriscissus* SMNS 81961; **D**, adult *S. quadriscissus* SMNS 80234. **Abbreviations**: **bof**, basioccipital facet; **ds**, dorsum sellae; **icf**, internal carotid foramen; **pas**, parasphenoid; **ptf**, pterygoid facet.
