# Supplementary material for: Ontogenetic variation in the skull of Stenopterygius quadriscissus with an emphasis on prenatal development
Source: Sci Rep. 2022 Feb 1;12:1707. doi: 10.1038/s41598-022-05540-0 (PMC8807662; doi:10.1038/s41598-022-05540-0)
Supplement: Supplementary file 2 — Supplementary Information 2. [file 41598_2022_5540_MOESM2_ESM.pdf]

Ontogenetic character states within the cranium of *Stenopterygius quadriscissus*

This document accompanies Table 1 and is an overview of the different ontogenetic states of all cranial elements of *Stenopterygius quadriscissus.* All dermatocranial observations are found in this work and braincase observations are taken from Miedema and Maxwell, 2019[1].

Antorbital rostrum

Premaxilla

0) Weak ossification; no premaxillary fossa
1) Premaxillary fossa present; ossification fibres apparent
2) Smooth surface

Nasal

0) Weak anterior ossification
1) Ossification fibres visible
2) Smooth texture

Maxilla

0) Ossification fibres visible/ossification onset
1) Smooth lateral texture, associated teeth relatively large
2) Smooth lateral texture, associated teeth relatively small

Circumorbital area

Lacrimal

0) Ossification fibres visible, no distinct medial depression
1) Distinct medial depression without anterior delination; ventral margin straight
2) Medial depression distinctly delineated anteriorly; ventral margin sometimes irregular

Jugal

0) Distinctly lunate/parabolic shape, small posterior bulge
1) In between lunate and more angular; distinct posterior bulge
2) Posterior bulge in line with the horizontal ramus; jugal angular in shape

Prefrontal

0) Onset ossification, no prominent orbital roof
1) Ossification fibres visible; very prominent orbital roof
2) Smooth texture orbital roof
3) Area of facets proportionally large compared to orbital roof

Postorbital

0) Ossification onset, no distinct facets
1) Ossification fibres visible, facets apparent
2) Smooth texture

3) Wide midsection

Postfrontal

0) Ossification onset, medial head present
1) Ossification fibres visible, orbital roof distinctly present
2) Smooth texture dorsally

Cheek area

Quadratojugal

0) Ossification fibres visible, triangular anterior edge, no distinct facets except for the quadrate facet
1) Smooth texture, facets developed

Squamosal

0) Ossification fibres visible
1) Smooth surface, obtuse angle between posterior and dorsal edge
2) Angle between posterior edge and dorsal edge approaching 90 degrees

Supratemporal

0) Ossification onset
1) Ossification fibres visible, facets relatively large
2) Smooth texture
3) Broadened lateral side, facets less prominent

Skull roof

Frontal

0) Weak ossification
1) Ossification fibres visible, large parietal foramen
2) Smooth surface texture, onset of development of process anterior to parietal foramen
3) Process anterior to parietal foramen developed, weak interfrontal midline ossification
4) Process anterior to parietal foramen robust, strong interfrontal midline ossification on occasion.

Parietal

0) Weak ossification
1) Ossification fibres visible, supratemporal ramus in-line with posterior edge of parietal
2) Ossification fibres visible, supratemporal ramus offset from posterior edge of parietal
3) Smooth dorsal surface, dorsal triangular plateau visible
4) Triangular plateau less apparent, development of a distinct posterior parietal shelf
5) Posterior parietal shelf delineated anteriorly by parietal ridge

Palatal region

Vomer

0) Weak ossification, especially anteriorly

1) Rugose texture still present, but anterior ossification strong
2) Main bone texture smooth, facets apparent

Palatine

0) Ossified rugose texture
1) Smooth bone texture

Pterygoid

0) Anterior fork present; palatine flange and lateral wing separate
1) No apparent anterior fork, anterolaterally directed lateral wing, confluence palatine flange/lateral wing with anterior ramus variable
2) palatine flange/lateral wing confluent with anterior ramus, directed laterally

Lower Jaw

Dentary

0) Dentary toothless
1) Teeth present but lateral groove only vaguely apparent
2) Lateral groove well apparent

Splenial

0) weak ossification, roughened surface texture
1) Ossified smooth surface texture
2) Symphyseal marks on anterior processes apparent

Surangular

0) Ossified, no clearly defined glenoid fossa
1) Ossified separate preglenoid and paracoronoid processes
2) Ossified confluent preglenoid and paracoronoid processes

Angular

0) Ossified, anterior margin less developed
1) Fully ossified

Prearticular

0) Unossified
1) Ossified with distinct fibres; ossification difference between angular facet and the rest of the element
2) Ossified with distinct fibres; ossification similar throughout
3) Ossified with smooth surface

Articular

0) Unossified
1) Ossified with no distinct glenoid and prearticular facets
2) Distinct facets present
3) Medial surface divided by a distinct midline

Hyoid apparatus

0) Unossified hyoid elements
1) Unossified basihyoid, ossified CB1
2) Ossified CB1 and basihyoid

Braincase

Quadrate

0) Unossified
1) Ossified, roughened texture; stapes facet medial
2) Smooth texture; quadrate condyle apparent
3) Stapes facet placed ventromedially
4) Lateral curvature occipital lamella; quadrate condyle often apparent as having two distinct bosses

Basioccipital

0) Anterior notochord pit; rugose texture extracondylar area
1) Smooth texture extracondylar area; but rugose on the condyle
2) Elongated basioccipital peg; exoccipital facets apparent, smooth texture on condyle
3) Distinct surrounding ridges and rugose texture of the foramen magnum floor
4) Bulbous, round basioccipital peg; opisthotic facets apparent

Parabasisphenoid

0) Ossified; triangular in dorsal view; parasphenoid fused to basisphenoid
1) Relatively quadrangular open dorsum sellae; possible full separation of the two canals of the internal carotid foramen
2) Closed dorsum sellae; large midline cleavage basioccipital facet; quadrangular outline; internal carotid foramen internally divided by a ridge
3) More winglike in ventral view due to the development of the basipterygoid processes
4) Reversal to more quadrangular morphology; internal dividing ridge of the internal carotid foramen often not apparent or vaguely apparent

Exoccipital

0) Unossified
1) Ossified, apparent hypoglossal foramen

Stapes

0) Unossified
1) Ossified rugose shaft and medial head
2) Smooth texture shaft

Opisthotic

0) Unossified
1) Ossified, distinct semicircular canal impressions
2) Short thickened or elongated paroccipital process

3) Distinct process visible posteriorly caused by the bulging of the posterior vertical semicircular canal.

Prootic

0) Unossified
1) Round semircircular canal impressions, impressions of vertical and horizontal semicircular canal not the same size
2) Semicircular canal impressions round and similar in size; small dividing ridge divides these impressions dorsally
3) Dividing ridge still apparent; semircircular canal impressions more triangular in shape
4) Dividing ridge vaguely apparent or absent

Supraoccipital

0) Unossified
1) Ossified quadrangular lateral edges; small triangular foramen magnum contribution
2) Round lateral edges; small round foramen magnum contribution
3) Large flattened dorsal margin; large round foramen magnum contribution
4) Round dorsal margin; lateroventral direction of exoccipital facets on occassion

1. Miedema, F. & Maxwell, E. E. Ontogeny of the braincase in Stenopterygius (Reptilia , Ichthyosauria) from the Lower Jurassic of Germany. *J. Vertebr. Paleontol.* **39**, (2019).
