# Supplementary material for: Ontogenetic variation in the skull of Stenopterygius quadriscissus with an emphasis on prenatal development
Source: Sci Rep. 2022 Feb 1;12:1707. doi: 10.1038/s41598-022-05540-0 (PMC8807662; doi:10.1038/s41598-022-05540-0)
Supplement: Supplementary file 3 — Supplementary Legends. [file 41598_2022_5540_MOESM3_ESM.pdf]

Table S1

Title: Ontogenetic character scores for the studied SMNS specimens.

Legend: Studied specimens are placed in their respective ontogenetic stage on the basis of characters of cranial ossification and morphology. Character states are discussed in supplemental file 2
