# Supplementary figures and images for: Ontogenetic variation in the skull of Stenopterygius quadriscissus with an emphasis on prenatal development
Source: Sci Rep. 2022 Feb 1;12:1707. doi: 10.1038/s41598-022-05540-0 (PMC8807662; doi:10.1038/s41598-022-05540-0)

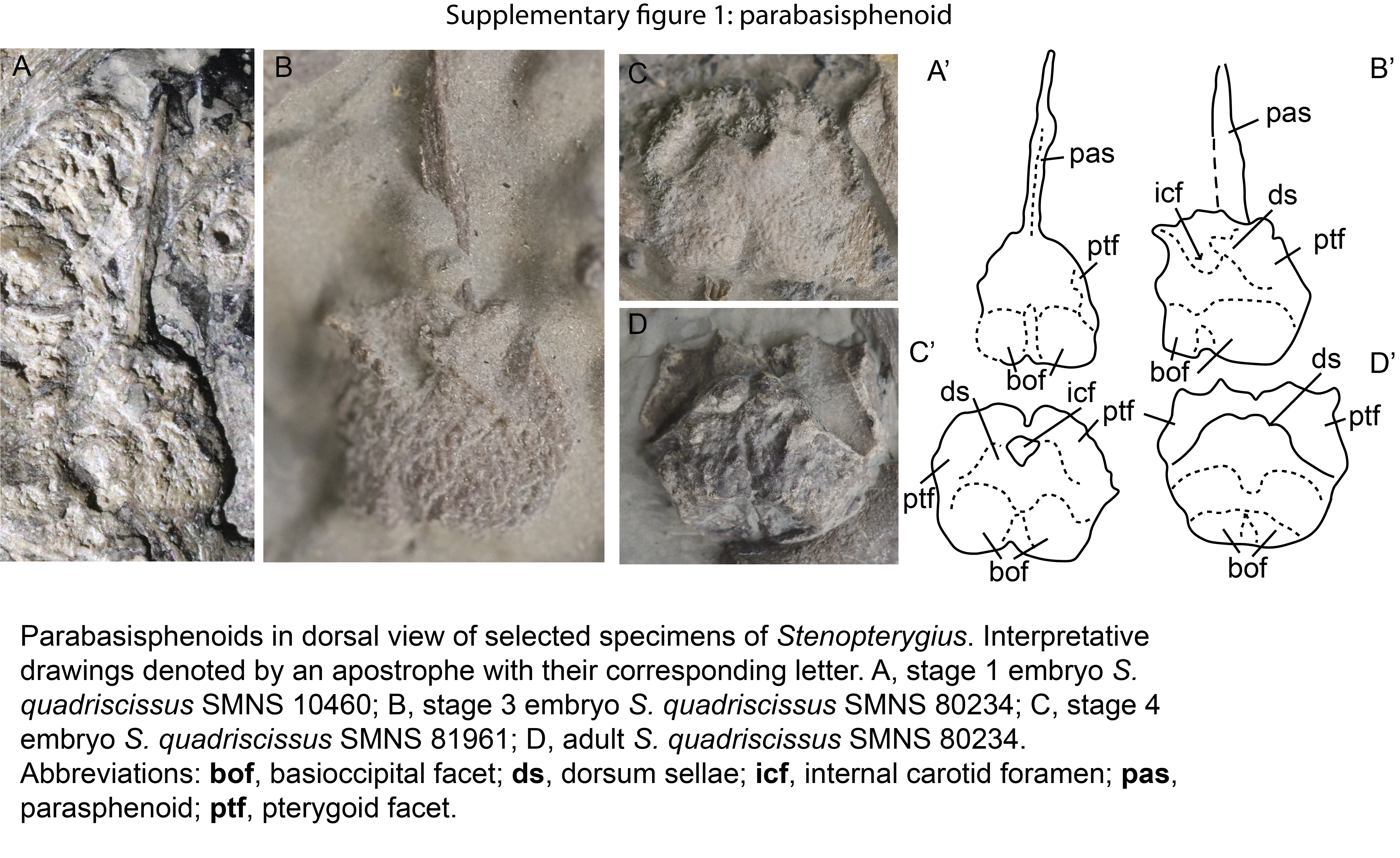

Supplement: Supplementary file 5 — Supplementary Figure 1. [file 41598_2022_5540_MOESM5_ESM.tif]

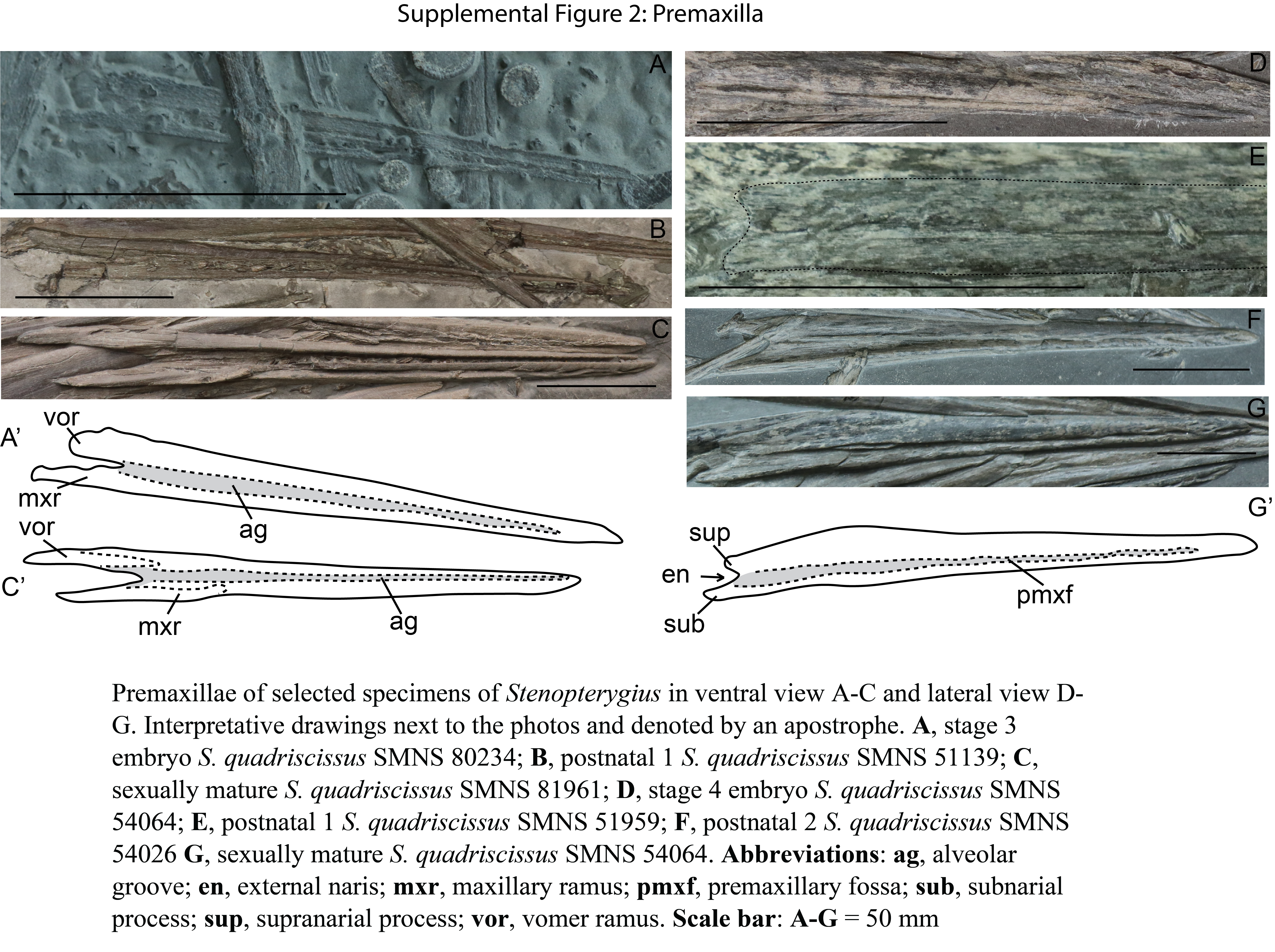

Supplement: Supplementary file 6 — Supplementary Figure 2. [file 41598_2022_5540_MOESM6_ESM.tif]

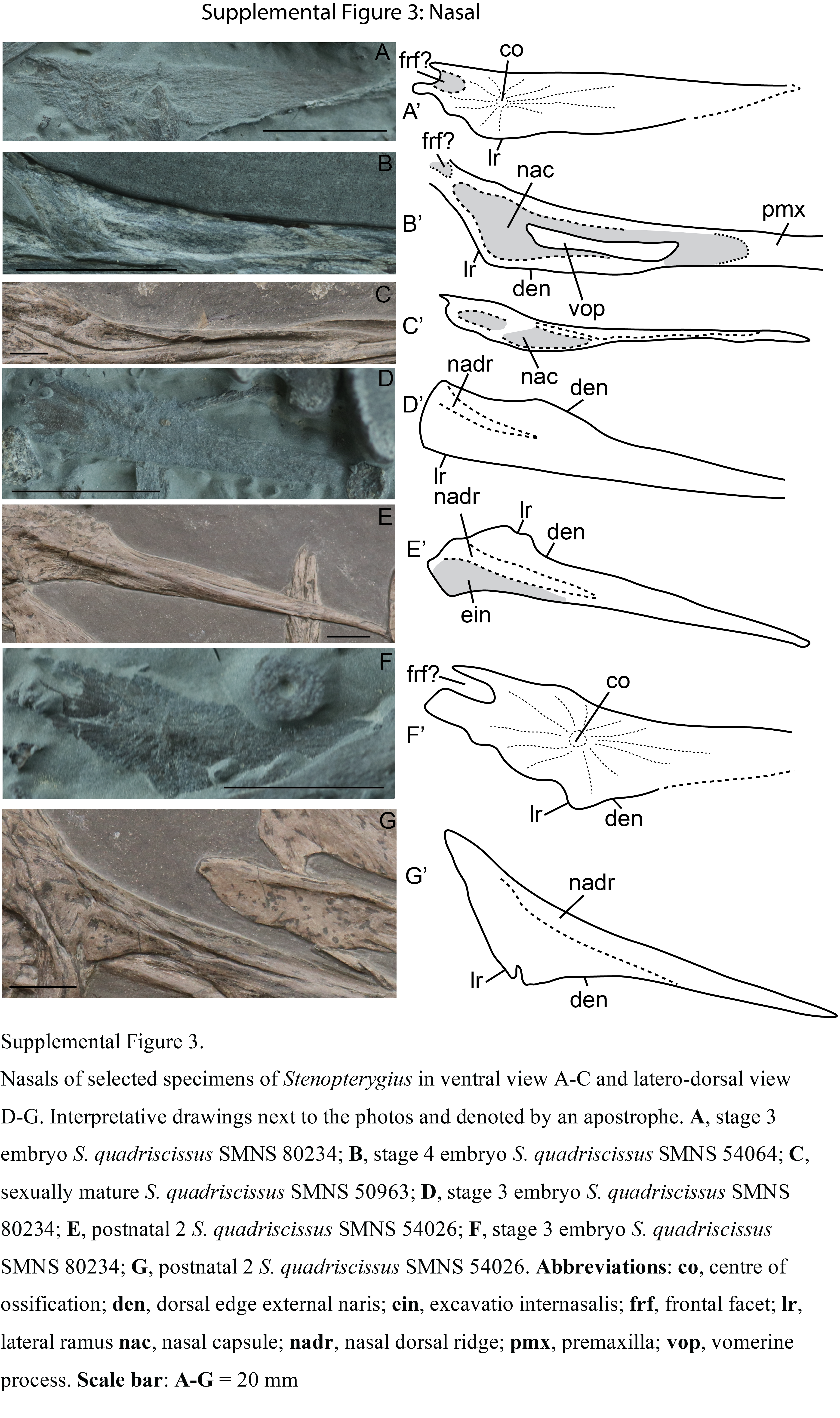

Supplement: Supplementary file 7 — Supplementary Figure 3. [file 41598_2022_5540_MOESM7_ESM.tif]

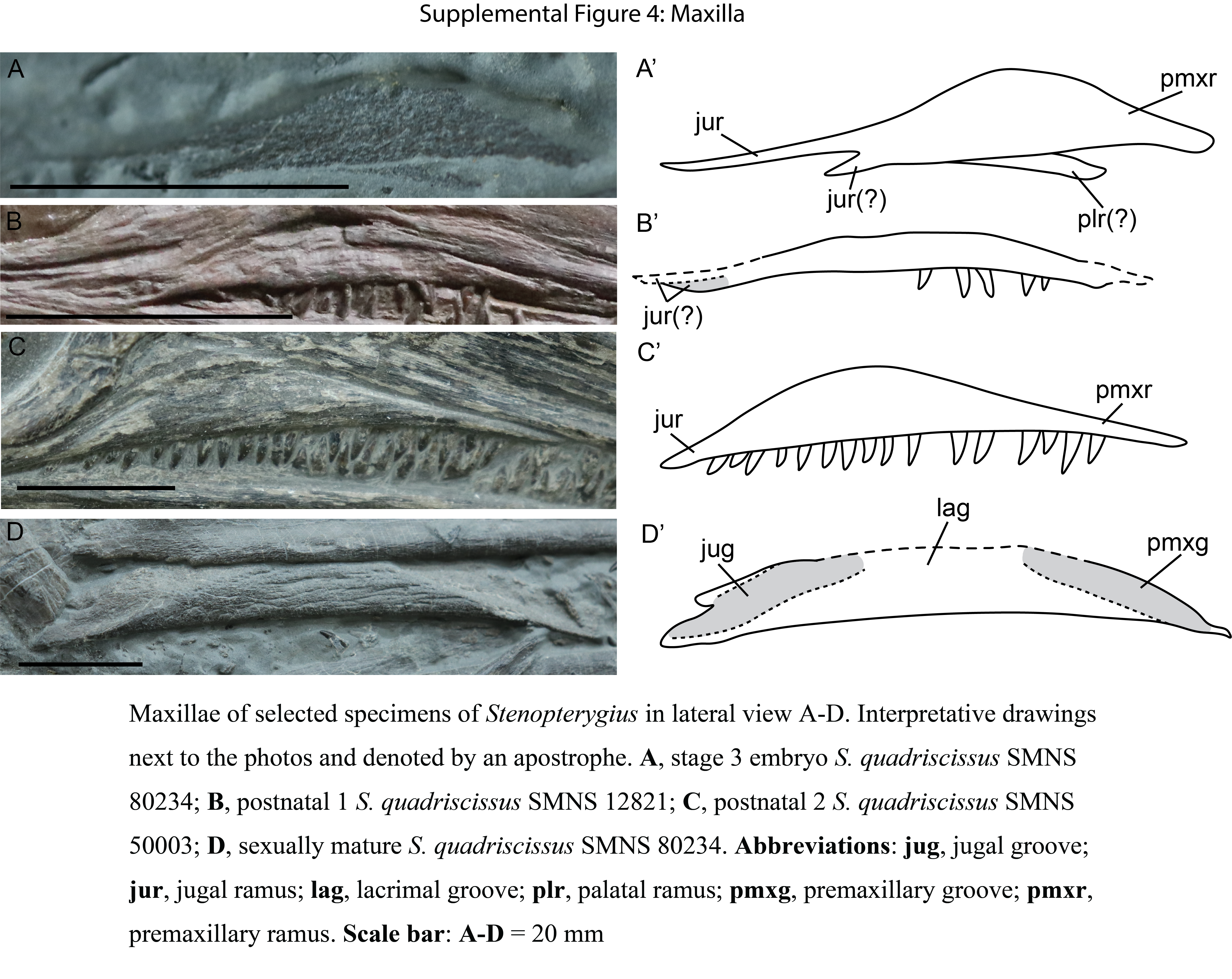

Supplement: Supplementary file 8 — Supplementary Figure 4. [file 41598_2022_5540_MOESM8_ESM.tif]

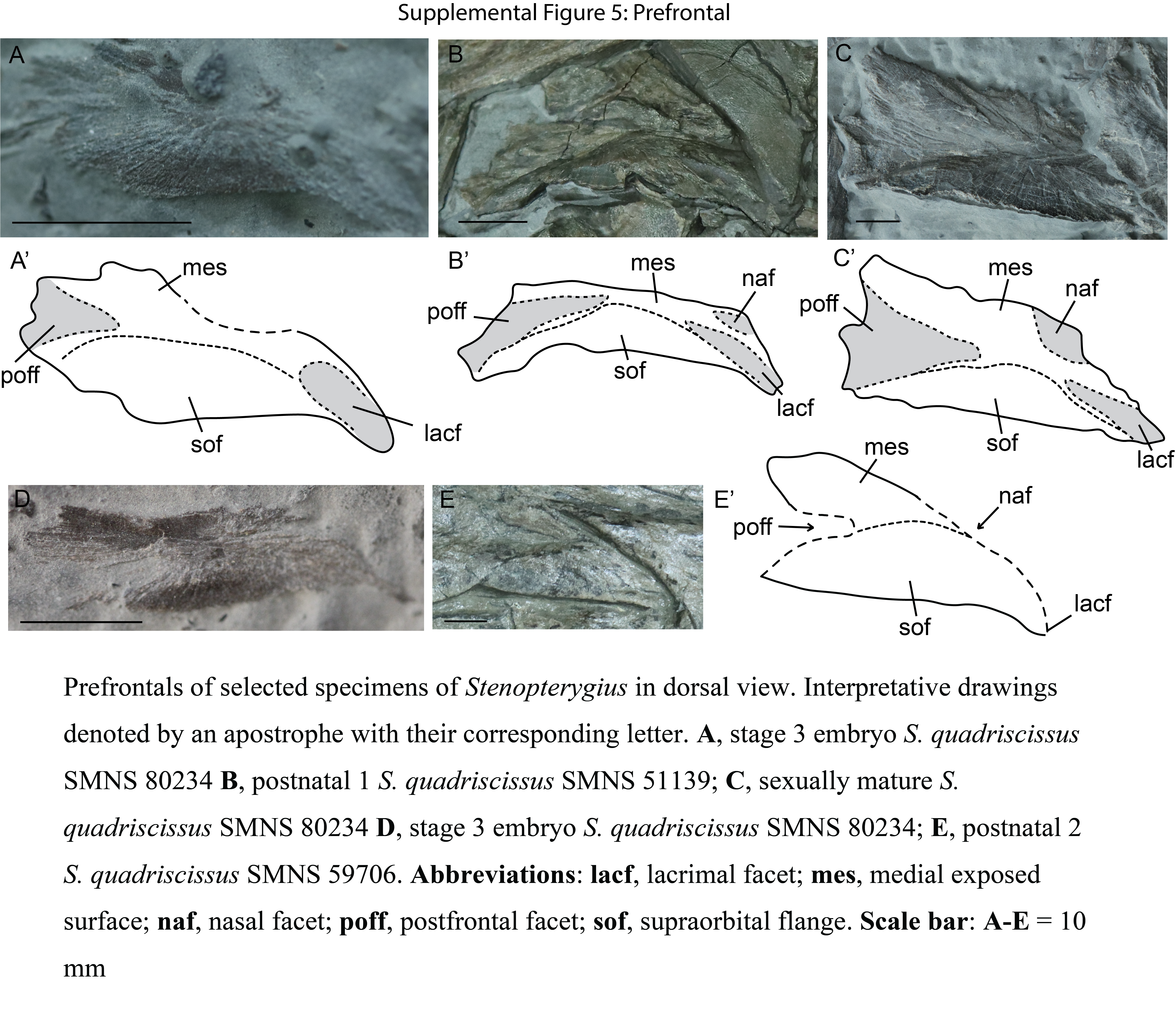

Supplement: Supplementary file 9 — Supplementary Figure 5. [file 41598_2022_5540_MOESM9_ESM.tif]

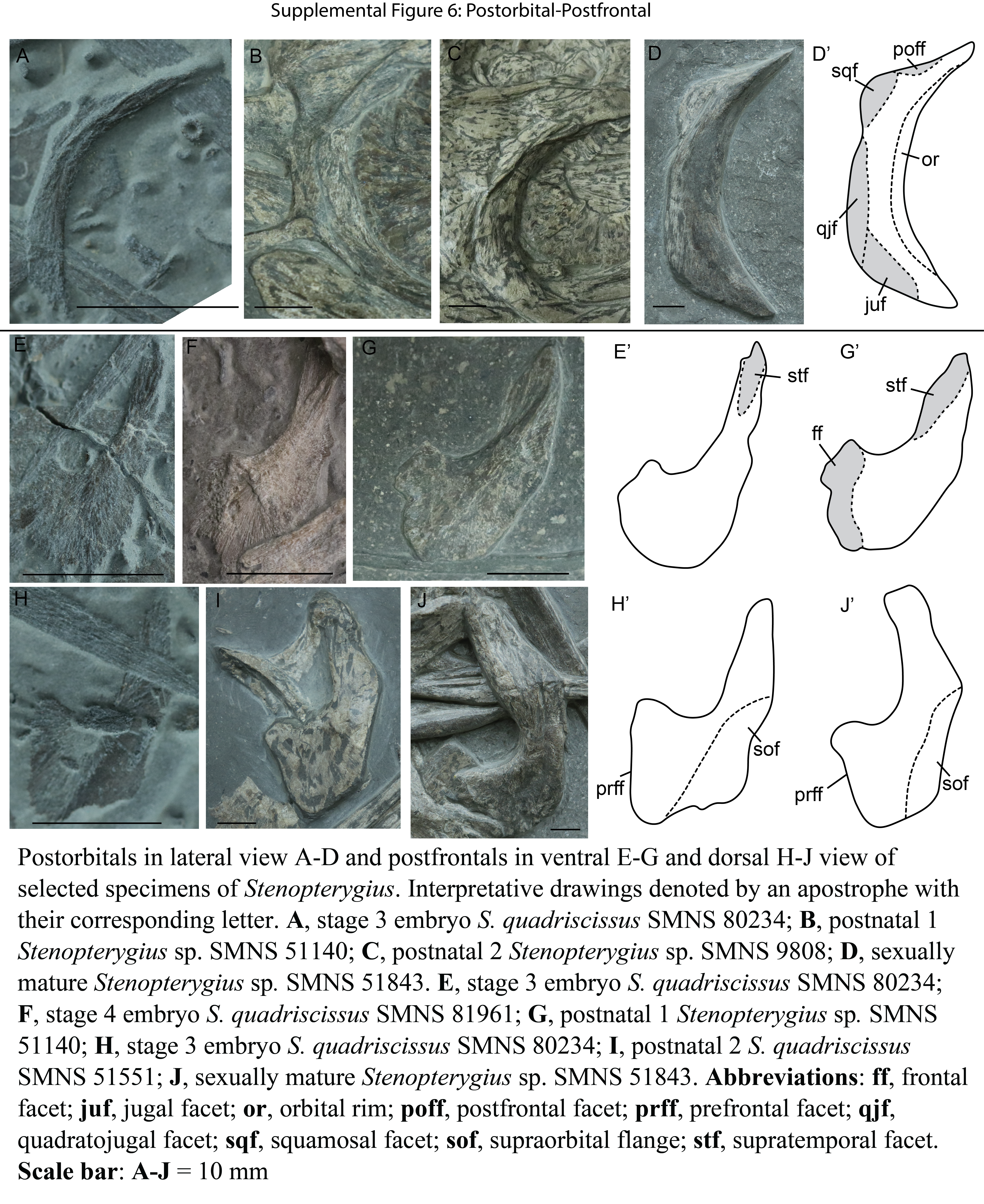

Supplement: Supplementary file 10 — Supplementary Figure 6. [file 41598_2022_5540_MOESM10_ESM.tif]

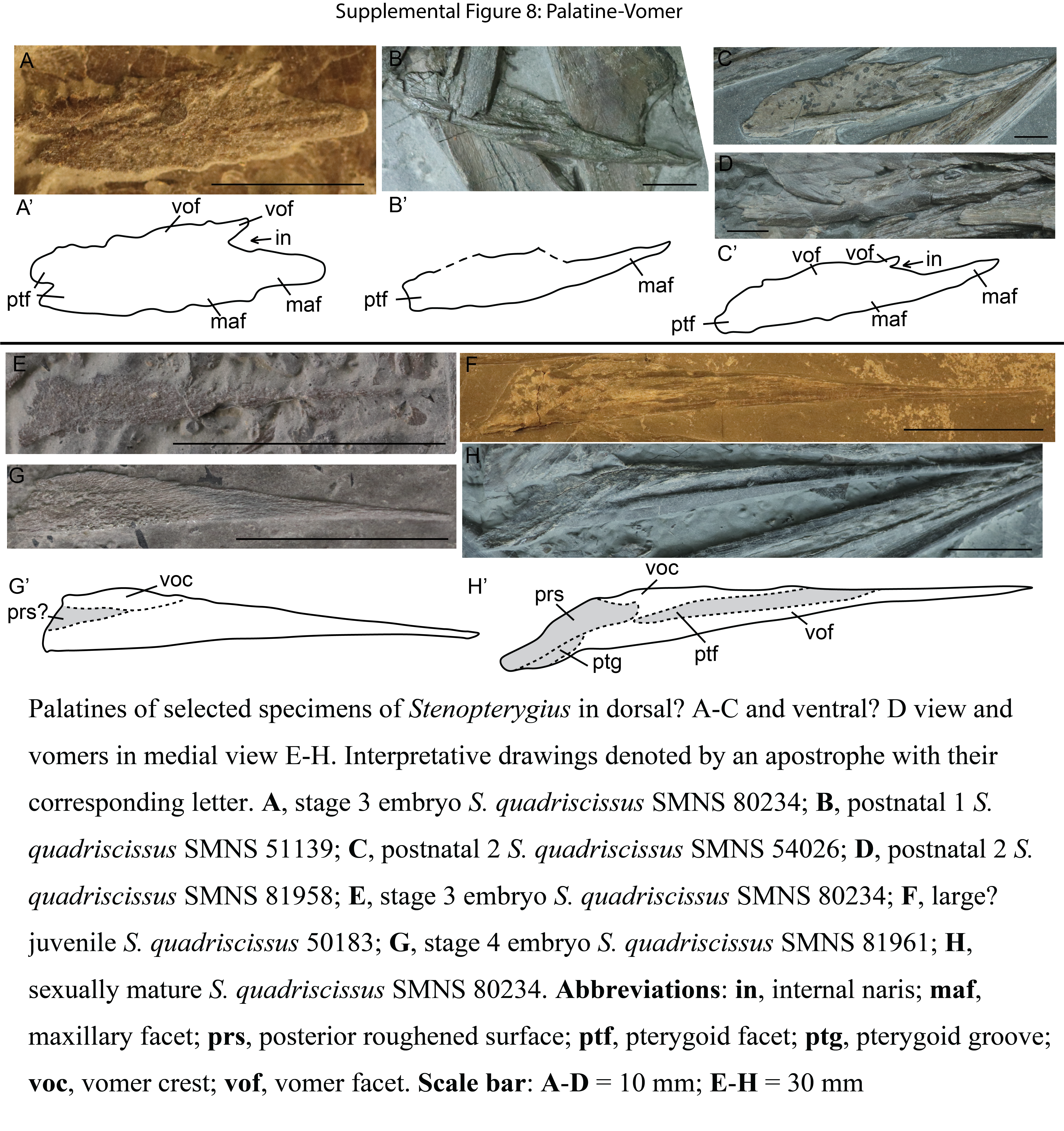

Supplement: Supplementary file 12 — Supplementary Figure 8. [file 41598_2022_5540_MOESM12_ESM.tif]

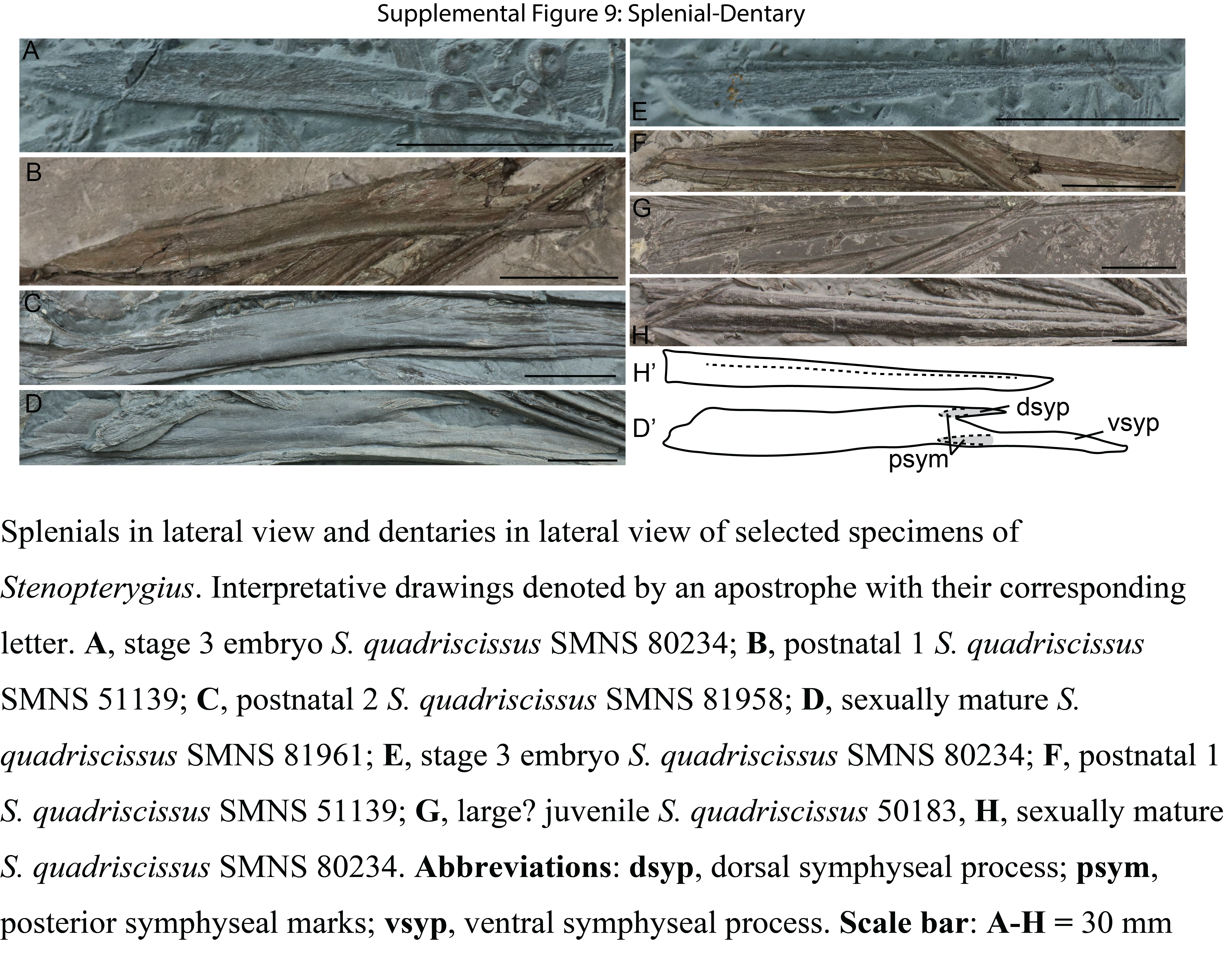

Supplement: Supplementary file 13 — Supplementary Figure 9. [file 41598_2022_5540_MOESM13_ESM.tif]

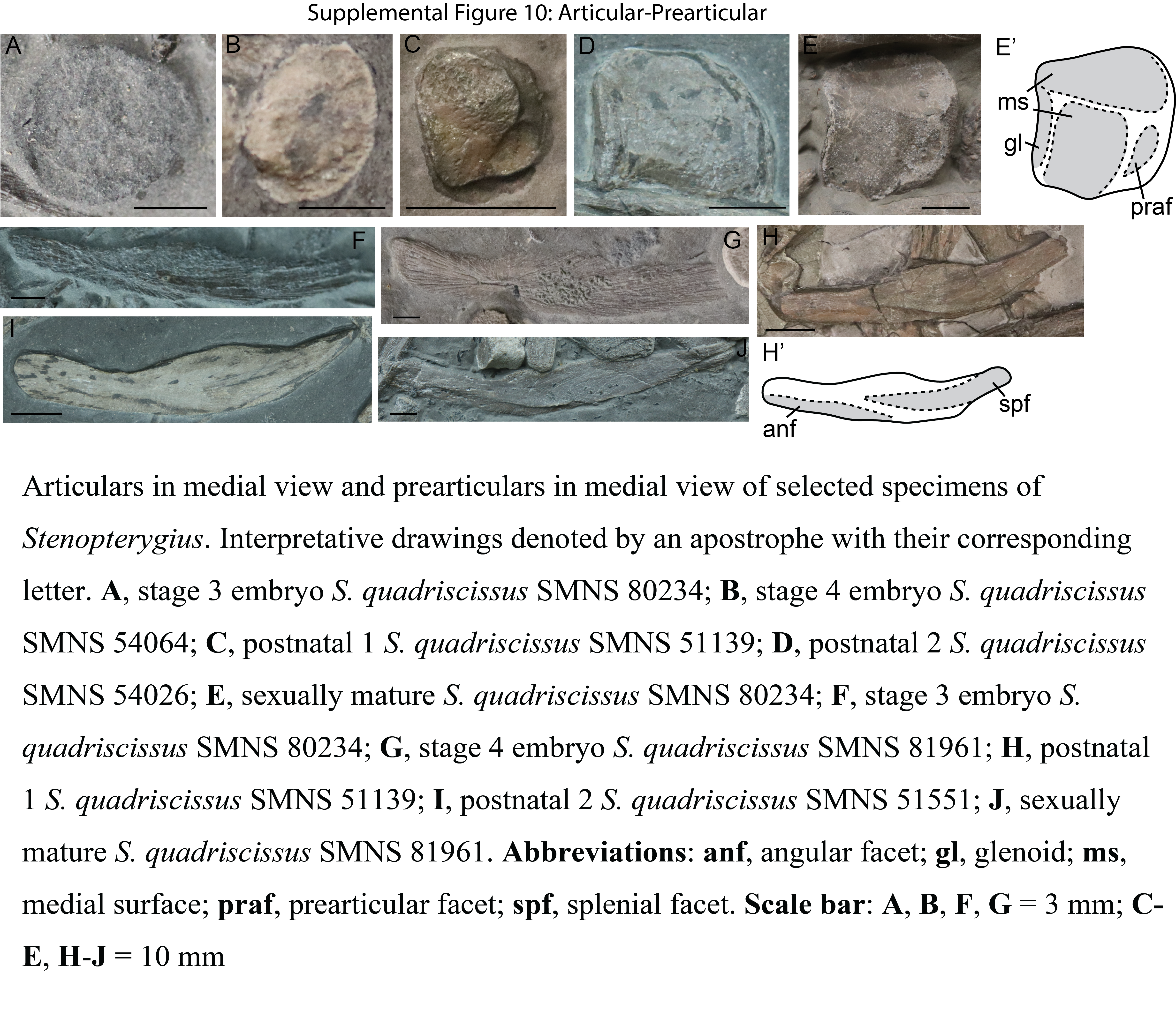

Supplement: Supplementary file 14 — Supplementary Figure 10. [file 41598_2022_5540_MOESM14_ESM.tif]
